# Sleep and obesity among children: A systematic review of multiple sleep dimensions

**DOI:** 10.1111/ijpo.12619

**Published:** 2020-02-18

**Authors:** Bridget Morrissey, Elsie Taveras, Steven Allender, Claudia Strugnell

**Affiliations:** ^1^ Global Obesity Centre Deakin University Geelong Australia; ^2^ Department of Pediatrics Massachusetts General Hospital for Children Massachusetts

**Keywords:** children, obesity, overweight, sleep, sleep dimensions

## Abstract

The objectives were to systematically investigate the multiple dimensions of sleep and their association with overweight or obesity among primary school‐aged children. CINHAL, PsycINFO, SPORTDiscus, Medline, Cochrane, Embase, and PubMed databases were searched for papers reporting on an association between children's sleep and weight status. Studies on clinical populations, published in languages other than English, without objectively measured weight status, or where weight status was reported outside the outlined age bracket (5‐13 years) were excluded. A total of 34  248 citations were extracted from our systematic search protocol, of which 112 were included for detailed review. Compared with sleep duration, of which 86/103 articles found a significant inverse association between sleep duration and measured weight status, few studies examined other dimensions of sleep, such as quality, efficiency and bed/wake times, and relationship with weight status. Where studies existed, variation in defining and measurement of these dimensions restricted comparison and potentially influenced discrepancies across results. Overall, the findings of this review warrant the need for further research of the outlined dimensions of sleep. Future research would benefit from clarity on definitions across the different dimensions, along with the use of valid and reliable tools.

**Abbreviations**
BMI: body mass index
BT: bed time
OR: odds ratio
Ow/Ob: overweight and obesity
PSG: polysomnography
SMP: sleep mid‐point
SWP: sleep wake pattern
WT: wake time

## 1 INTRODUCTION

There is growing evidence internationally that declines in the duration of sleep children obtain is inversely associated with overweight and obesity (Ow/Ob).^1^, ^2^, ^3^, ^4^ Studies have shown that where children were classified as sleeping for shorter or insufficient durations they had significantly increased odds of being affected by overweight/obesity, compared with those who slept for sufficient durations.^5^, ^6^ The proposed etiology of this association suggests that insufficient sleep leads to an energy imbalance via altered hormone regulation, reducing physical activity levels, increasing sedentary time, and a higher caloric intake.^6^, ^7^, ^8^ While several reviews have examined the available literature on the link between sleep duration and rates of overweight and obesity among children,^9^, ^10^, ^11^, ^12^, ^13^, ^14^ there is argument that these overlook more nuanced and potentially important dimensions of children's sleep that might influence this association.

Buysse critiqued the reliance on duration as a measure of sleep, arguing that the dimensions of children's sleep such as efficiency, quality, and sleep timing (onset/off‐set) should also be considered as part of the sleep‐obesity relationship.^15^ Definitions and categorization of these dimensions differ slightly across studies; however, as guided by definitions from the previous literature,^15^, ^16^, ^17^ these four sleep dimensions can be defined as: *Sleep duration:* the quantity/length of sleep time obtained; *Sleep quality*: objectively measured architecture of sleep (adequacy of time spent in the different sleep wave cycles), or subjectively reported satisfaction/perceived problems with sleep; *Sleep efficiency*: a measure of sleep continuity, incorporating the ease to initiate (sleep latency) and maintain sleep (minimal wake episodes) in an efficient manner, or the percentage of sleep time achieved between bed and wake times; and *Sleep timing*: the placement of sleep within the 24‐hours of the day, including factors such as bed/wake times.

Beyond the prominent focus of children's sleep duration on the sleep‐obesity association of previous reviews, 9, 10, 11, 12, 13, 14 there is emerging literature for each of these additional dimensions. For example Jarrin et al studied 240 Canadian children and adolescents (aged 8‐17 years) and found that, independent of self‐reported sleep duration, children with delayed sleep timing (late to bed and late to wake) related to a higher risk of being affected by overweight and obesity relative to early to bed/wake counterparts.^18^ An Australian study found later bedtimes were linked with higher body mass index (BMI) z‐scores and lower diet quality scores regardless of self‐reported wake time.^19^ Sleep quality and sleep efficiency have also been highlighted as important dimensions of sleep associated with overweight and obesity risk among children.^20^, ^21^, ^22^ Lui et al used polysomnography (PSG) to analyze sleep quality by recording stages of sleep among a sample of American children and adolescents (7‐17 years). It is suggested that nonrapid eye movement (REM) sleep could be an important stage of sleep for endocrine and metabolic regulation as reduced REM was associated with higher BMI z‐scores.^23^ Furthermore, the authors also reported that higher sleep efficiency (determined by the percentage of time spent asleep between sleep onset to wake time, measured via PSG) was a significant dimension of sleep associated with reduced risk of overweight and obesity.^23^ While PSG is considered the gold standard for measuring multiple sleep components, it is quite invasive and costly and therefore not always practical for use in larger samples.^24^ Self‐reported proxies for sleep quality and efficiency have therefore often been used, with results appearing to support those from more objective measures. Studies have reported that those with lower self‐perceived sleep quality (ie, less likely to report sleeping “well”) or lower perceived sleep efficiency (reported issues around waking up during the night or issues falling asleep) are more likely to experiences poorer weight status outcomes.^25^

With emerging empirical evidence of associations between the specific dimensions of sleep and weight status, a more nuanced understanding of these associations is now possible and needed to adequately inform future obesity prevention initiatives.^11^, ^12^ A systematic understanding of the current conceptions and measurements of the dimensions of sleep is needed; and an examination of variability of results across the different dimensions of sleep will help determine the importance of these on the sleep‐obesity association, among population samples.

As sleep requirements notably vary across the life span, with quite considerable differences in optimal sleep duration recommendations for primary school aged children (5‐13 years; 9‐11 hours per night) compared with those for adolescents (14‐18 years; 8‐10 hours per night), the association between sleep habits and obesity needs to be unpacked across the age groups.^26^, ^27^ Furthermore, there is a particular need to better understand the etiology of obesity among early primary school‐aged children. Data from the National Child Measurement Program in England found that of children with obesity at the beginning of primary school only 10% were of a healthy weight by the end of primary school, while more than two thirds remained in the category of being with obesity or severe obesity.^26^ Therefore, with strong evidence for the tracking of obesity and health behaviors from this age through to adolescence and beyond, 26, 27, 28, 29 along with the differences in sleep needs, there is a need to better understand the sleep‐obesity nexus of this particular at risk age group. This article presents a systematic review of the peer‐reviewed literature with data on the association between different dimensions of sleep and weight status among primary school‐aged children (5‐13 years old).

## 2 METHOD

### 2.1 Literature search

Following the Preferred Reporting Items for Systematic Reviews and Meta‐Analyses (PRISMA) review process, a systematic search was conducted across CINHAL, PsycINFO and SPORTDiscus (via EBSCOhost), Medline, Cochrane, Embase, and PubMed databases. A search strategy (available in Table S1) was used to extract literature published up until March 2018. Our search strategy was informed by previous systematic reviews of sleep and weight status of children,^9^, ^10^, ^11^, ^12^, ^13^, ^14^ including all appropriate search terms and additional terms for dimensions of sleep. The search strategy was adapted to search each database for original research articles published in English and in peer‐reviewed journals.

### 2.2 Inclusion/exclusion criteria

Included studies were restricted to (a) peer‐reviewed original research; (b) contain some measure of sleep; (c) had objectively measured weight status; (d) report on the association between the two variables (sleep and weight status); (e) participants were primary school aged children (aged 5‐13 years); and (f) sample was nonclinical/free‐living population. Reviews, meta‐analyses, dissertations, expert opinions, conference abstracts, unpublished studies, and studies published in a language other than English were excluded from the review. Studies were also excluded if the main outcome variable (children's weight status) was reported outside the age bracket (5‐13 years).

### 2.3 Recording and synthesis of findings

For each included article, the two reviewers (B.M. and C.S.) independently utilized a developed data extraction tool (available in Table S2) to obtain relevant information for each study (eg, study design, sample characteristics, dimension[s] of sleep analyzed, association of each dimension, and the main findings). The study quality was also assessed for each article utilizing an adjusted version of the Newcastle‐Ottawa Scale (NOS),^30^, ^31^ with a 10‐point star scale (10 as the highest quality) to critic studies based on the selection of the study groups (two points), the comparability of the groups (three points), and the ascertainment of either the exposure or outcome of interest (five points).

The two data sets were then cross checked and discrepancies amended, using a third reviewer (S.A.) if required. Once included in the review, a narrative synthesis of the data was conducted. Studies were grouped by dimension of sleep and then measurement type categories, enabling comparison of results and measurements across and within each of the different dimensions of sleep.

## 3 RESULTS

**Figure 1:**
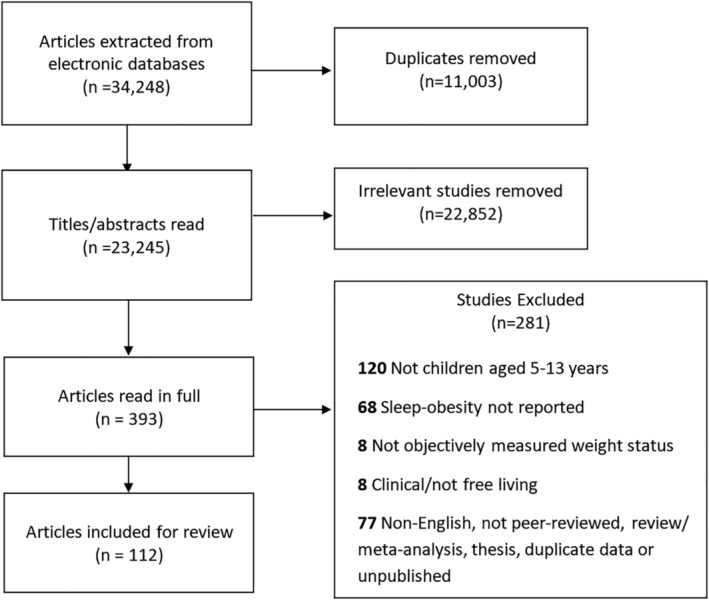
Flow chart of article selection process

A total of 34  248 citations were extracted from the outlined databases using the key search terms, and duplicate citations (n = 11  001) were removed (see Figure 1). Following the inclusion/exclusion criteria, abstracts were screened and a total of 22  852 irrelevant articles were excluded. The remaining 393 articles were read in full, with 281 excluded: 120 did not meet the age criteria (5‐13 years); 8 were of clinical or nonfree‐living population groups; 8 did not measure weight status objectively; 68 did not analyze the association between sleep‐obesity; and 77 were excluded either due to being non‐English, not peer‐reviewed, review/meta‐analysis, duplicate data, or a conference abstract/other unpublished data. This left 112 articles deemed relevant and included in the analysis (Table 1).^8^, ^20^, ^21^, ^22^, ^32^, ^33^, ^34^, ^35^, ^36^, ^37^, ^38^, ^39^, ^40^, ^41^, ^42^, ^43^, ^44^, ^45^, ^46^, ^47^, ^48^, ^49^, ^50^, ^51^, ^52^, ^53^, ^54^, ^55^, ^56^, ^57^, ^58^, ^59^, ^60^, ^61^, ^62^, ^63^, ^64^, ^65^, ^66^, ^67^, ^68^, ^69^, ^70^, ^71^, ^72^, ^73^, ^74^, ^75^, ^76^, ^77^, ^78^, ^79^, ^80^, ^81^, ^82^, ^83^, ^84^, ^85^, ^86^, ^87^, ^88^, ^89^, ^90^, ^91^, ^92^, ^93^, ^94^, ^95^, ^96^, ^97^, ^98^, ^99^, ^100^, ^101^, ^102^, ^103^, ^104^, ^105^, ^106^, ^107^, ^108^, ^109^, ^110^, ^111^, ^112^, ^115^, ^116^, ^117^, ^118^, ^119^, ^120^, ^121^, ^122^, ^123^, ^124^, ^125^, ^126^, ^127^, ^128^, ^129^, ^130^, ^131^, ^132^, ^133^, ^134^, ^135^, ^136^, ^137^, ^138^, ^139^, ^140^, ^141^

**Table 1:** Study characteristics and main results^a^

First author (year)	Sample size; country; study design	Age group	Measure of sleep	Measure of weight status	Report association				Study quality
					Sleep duration	Sleep efficiency	Sleep quality (low to high)	Sleep timing	
Agras et al (2004)^32^	150; USA; LT	9.5 y (from birth)	Parent report	BMI >85th percentile = OW/OB	✓ NEG				3
Alamian et al (2016)^33^	895; USA; LT	11 ± 2	Parent report	BMI z (CDC)			✓ NEG		7
Alqaderi et al (2017)^34^	6316; Kuwait; LT	12 (from 10)	Self‐report	WC				✓ POS	6
Altenburg et al (2013)^35^	5757; Belgium, Greece, Hungary, The Netherlands, Norway, Slovenia and Spain; CS	10‐12 y	Parent report	BMI (IOTF); WC	✓ NEG				10
Amigo et al (2014)^36^	291; Spain; CS	9‐10 y	Self‐report	BMI (IOTF)	✓ NEG			✓ POS (BT)	6
Anderson et al (2017)^37^	10 995; UK; LT	11 (from 3)	Parent report	BMI z (IOTF)				✓ SIG (BT consistency)	8
Anujuo et al (2016)^38^	2384; Amsterdam; CS	5	Parent report	BMI z (IOTF)	NS				6
Arora and Taheri (2015)^39^	511; UK; CS	11‐13 y	Accelerometry	BMI‐z	✓ NEG	NS (%)			8
Bagley and El‐Sheikh (2013)^40^	228; USA; CS	9‐12 y	Accelerometry	BMI z (CDC)	✓ NEG	✓ NEG (%)			7
Bagley and El‐Sheikh (2014)^20^	235; USA; CS	8‐10 y	Accelerometry	BMI z (CDC)	✓ NEG	✓ NEG (%) POS (LWE)			7
Barlett et al (2012)^41^	1156; USA; LT	6‐12 y	Self‐report	BMI z (CDC)	✓ NEG (CS and L)				6
Bayer et al (2009)^42^	7767; Germany; CS	3‐10 y	Parent report	BMI z (IOTF); BF% (skin folds‐KFA)	✓ NEG				3
Bell and Zimmerman (2010)^43^	1108; USA; LT	5‐8 y (from 0 to 4)	Parent report	BMI z (CDC)	✓ NEG (CS and L)				7
Berentzen et al (2014)^44^	1481; Netherlands; CS	11‐12 y	Self‐report	BMI z (IOTF); WC	✓ NEG	NS (night awakenings)	NS	NS (SWP)	5
Busto‐Zapico et al (2014)^45^	291; Spain; CS	9‐10 y	Parent report	BMI z (ITOF)	NS			✓ POS (BT)	5
Cameron et al (2013)^46^	7234; Europe (Belgium, Greece, Hungary, the Netherlands, Norway, Slovenia and Spain); CS	10‐12 y	Parent report	BMI z (IOTF); WC	✓ NEG				6
Cao et al (2015)^47^	8760, China, CS	6‐12	Self‐report	BMI	✓ NEG (girls), POS (boys)				6
Carrillo‐Larco et al (2014)^48^	1929; Ethiopia, India, Peru and Vietnam; CS	7‐8 y	Parent report	BMI z (IOTF)	NS				5
Carter et al (2011)^49^	202; NZ; LT	7 y (from 3 and 5)	Accelerometry	BMI; BF% (bioelectrical impedance)	✓ NEG (CS and L)				10
Casazza et al (2011)^50^	104 (subsample); UK; CS	7‐12 y	Parent report	BF% (DXA); BMI z (CDC)	NS				6
Cassimos et al (2011)^51^	353; Greece; CS	11‐12 y	Self‐report	BMI z (IOTF)	NS			NS (BT)	4
Chahal et al (2013)^52^	3398; Canada; CS	10‐11 y	Parent report	BMI z (IOTF)	NEG				8
Chaput and Tremblay (2006)^53^	422; Canada; CS	5‐10 y	Parent report	BMI z (IOTF); WC	✓ NEG				6
Colley et al (2012)^54^	878; Canada; CS	6‐11 y	Accelerometer (nonwear time) and; parent report	BMI; WC	✓ NEG (accel only)				8
Combs et al (2016)^55^	348, USA, CS (LT out of age range)	7.6‐10.1	Parent report	BMI z (CDC)	✓ NEG (weekday only, NS weekend)			✓ POS (BT) NS (WT)	5
De Jong et al (2012)^56^	840; Netherlands; CS	9‐13 (subsample)	Parent report	BMI z (IOTF); WC	✓ NEG				7
Del Pozo‐Cruz et al (2017)^57^	Subsample of 1812, New Zealand, CS	5‐9	Self‐report	BMI	✓ NEG				6
Diethelm et al (2011)^58^	481; Germany; LT	7 y (from 2)	Parent report	BMI z (IOTF); BF% (skin fold‐Deurenberg); FMI; FFMI	✓ NEG				7
Drescher et al (2011).^59^	319; USA; CS	10‐13 y	Parent report	BMI z (CDC); skin fold	✓ NEG				4
Duncan et al (2008)^60^	1229; NZ; CS	5‐11 y	Parent report	BMI; BF% (bioelectrical impedance)	✓ NEG				7
Duran and Haro (2016)^61^	1810; Chile; CS	6‐11	Parent report	BMI	✓ NEG				8
Eisenmann et al (2006)^62^	6324; Australia; CS	7‐13 y (subsample)	Self‐report	BMI z; WC	✓ NEG (boys Only)				8
Ekstedt et al (2013)^63^	1231; Sweden; CS	6‐10 y	Accelerometry	BMI z	✓ NEG	NS (%)		NS (BT; WT)	4
El‐Sheikh et al (2007)^64^	167; USA; CS	8‐9 y	Accelerometry	BMI z (CDC)	✓ NEG	✓ NEG (%)			7
El‐Sheikh et al (2014)^21^	269; USA; LT	9(T1), 10(T2), 11(T3)	Accelerometry (duration); self‐report (sleep problems)	BMI (CDC)	✓ NEG (CS and LT)		✓ POS (sleep problems)		6
Fernandez‐Mendoza et al (2014)^65^	327; USA; CS	5‐12 y	PSG (duration); Parent report (sleep problems)	BMI z (CDC); WC	✓ NEG	NS			6
Ferrari et al (2017)^66^	328, Brazil, CS	9‐o 11	Self‐report	BMI (WHO)	NS		NS		8
Firouzi et al (2013)^67^	183; Malaysia; CS	6‐12 y	Parent report	BMI z (WHO)	NS	NS (sleep onset)	✓ NEG (sleep disorder score)	NS (BT; WT)	5
García‐Hermoso et al (2017)^68^	395; Chile; CS	12‐13	Self‐report	BMI z (IOTF)	✓ NEG (girls only)		✓ NEG (girls only)		7
Gentile et al (2014)^69^	1323; USA; LT (only reports CS)	9 (±0.94) y	Self‐report	BMI	✓ NEG (CS)				6
Giovaninni et al (2014)^70^	370; Brazil; CS	6‐13 y	Parent report	BMI, waist and hip circumferences	✓ NEG				6
Gomes et al (2014)^71^	686; Portugal; CS	9‐11 y	Accelerometry	BMI z (IOTF)	NS				9
Harrex et al (2017)^72^	439, New Zealand, CS	9‐11	Accelerometry	BMI (WHO)				NS (SWP)	7
Hense et al (2011)^73^	4348 (subsample); Europe (Italy, Estonia, Cyprus, Belgium, Sweden, Hungary, Germany and Spain); CS	6‐9 y (subsample)	Parent report	BMIz (IOTF)	✓ NEG				8
Hiscock et al (2011)^74^	4464 (subsample); Australia; LT	6‐7 y (from 4 to 5)	Parent report	BMI z (CDC/IOTF)	✓ NEG (CS only)		NS		7
Hjorth et al (2014)^75^	785 CS; 708 LT; Denmark; LT	8‐11 y	Accelerometry (duration); parent (quality)	BMI z (WHO) Fat Mas Index (DXA)	✓ NEG (CS only)		NS		7
Ievers‐Landis et al (2008)^76^	819; USA; CS	8‐11 y	Parent report	BMI (CDC)	✓ NEG				6
Jiang et al (2014)^77^	1309; China; CS	10‐12	Parent report	BMI z; waist/height ratio; bf% (skin fold)	✓ NEG (girls only)				8
Jing Jing et al (2017)^78^	894, Hong Kong, CS	9‐12	Self‐report	BMI	✓ NEG		NS		6
Katzmarzyk et al (2015)^79^	6025, Australia, Brazil, Canada, China, Colombia, Finland, India, Kenya, Portugal, South Africa, United Kingdom, United States, CS	9‐11	Accelerometry	BMIz (WHO)	✓ NEG				7
Kelly et al (2016)^80^	16 936, UK, LT	11 (from 3 to 5 to 7)	Parent report	BMI				✓ NEG (non‐regular BT) POS (BT > 9 pm; LT)	6
Khan et al (2015)^82^	5560, Canada, CS	10‐11	Parent report	BMI z (IOTF)	✓ NEG			✓ POS	7
Khan et al (2017)^81^	2261, Canada, CS	10‐11	Parent report	BMI z (IOTF)	✓ NEG		✓ NEG		6
Kim et al (2012)^83^	936; Korea; CS	10‐11 y	Parent report	BMI z (2007 Korean National Growth Charts);	✓ NEG				7
Kong et al (2011)^84^	779 (the primary school sample); Hong Kong; CS	6‐14 y	Self‐report	BMI, WC	✓ NEG				8
Kovács et al (2015)^85^	8848, Estonia, Sweden, Germany, Belgium, Hungary, Italy, Spain and Cypru, CS	6‐9.9	Parent report	BMI z (IOTF)	✓ NEG				6
Krishnan et al (2017)^86^	643, New Zealand, CS	6	Accelerometry	BMI z (IOTF)	NS				8
Labree et al (2015)^87^	1943; Netherlands; CS	8‐9	Parent report	BMI z (IOTF)	✓ NEG				6
Larsen et al (2017)^88^	206; Netherlands; CS	7‐12	Parent report	BMI z	✓ NEG (boys only)				4
Laurson et al (2014)^89^	674; USA; CS	7‐12 y	Self‐report	BMI z (CDC)	✓ NEG (Boys Only)				6
Lee et al (2012)^90^	1504; South Korea; LT	7‐11 y (baseline)	Self‐report	BMI z (2007 growth chart for Korean children)	✓ NEG (LT only)				6
Lehto et al (2011)^91^	604; Finland; CS	9‐11 y	self‐report	WC and waist to height ratio	✓ NEG				8
Liu et al (2011)^92^	606; Canada; CS	11‐13 y	Parent report	BMI z (IOTF)	✓ NEG	✓ POS (Awakenings) NS (sleep onset)	✓ NEG (overall sleep problem score)		7
Lu et al (2015)^93^	2457, China, CS	7‐10	Self‐report	BMI	NS				3
Lumeng et al (2007)^94^	785; USA; LT	11.61 (±0.15) y (from 8 y)	Parent report	BMI z (National Center for Health Statistics norms)	✓ NEG (CS and LT)	NS (night awakenings)	NS (CHSQ score; CS and LT)	✓ POS (BT) NS (WT)	8
Magee et al (2013)^95^	1079; Australia; LT	10‐11 y (from 4 to 5)	Parent report	BMI z (IOTF) — three trajectories (early onset, late onset, and healthy)	✓ NEG (CS and LT)				8
Magee et al (2013)^96^	1833; Australia; CS	8‐9 y (from 6 to 7)	Parent report	BMI z (IOTF)	NS				6
Magee et al (2014)^8^	2984; Australia; LT	8‐9 y (from 4 to 5)	Parent report	BMI	✓ NEG				9
Martinez et al (2014)^98^	229; U.S.A; LT	10‐12 y (from 8 to 10)	Accelerometry	BMI z (CDC); waist‐to‐height ratio	✓ NEG				6
Martinez et al (2014)^97^	303; U.S.A; CS	8‐10 y	Accelerometer (actical) and parent report	BMI z (CDC)	✓ NEG (both Ob and Sub)				7
Martoni et al (2016)^99^	115, Italy, CS	10	Accelerometry	BMI z (IOTF)	✓ NEG			✓ POS (mid‐point)	5
McNeil et al (2015)^22^	515; Canada; CS	9‐11 y	Accelerometry	BMI z (IOTF); BF% (portable Tanita); WC	NS	✓ NEG (%)		NS (BT; WT; MP)	8
Meng et al (2012)^100^	6576; China; CS	7‐11 y	Self‐report	BMI, WC, BF%	✓ NEG				8
Miller (2011)^101^	11 400; USA; LT	11.23 y (from 6.23)	Parent Report	BMI	✓ NEG (CS only)				6
Morrissey et al (2016)^102^	298; Australia; CS	9‐13	Self‐report	BMI z (WHO)	✓ NEG				6
Munakata et al (2010)^103^	216; Japan; CS	9‐10 y	Self‐report	BMI; BF% (bioelectrical impedance)	✓ NEG				4
Ochiai et al (2012)^104^	3433; Japan; CS	9‐10 y	Parent report	BMI z (IOTF)	✓ NEG (Boys Only)			NS (BT; WT)	6
O'Dea et al (2012)^105^	939; Australia; CS (longitudinal out of age group)	7‐12 y	Self‐report	BMI z (IOTF)	✓ NEG (CS)				7
Ortega Anta et al (2013)^106^	7659; Spain; CS	6‐9 y	Parent report	BMI z (reference tables for Spanish children)	✓ NEG				6
Padez et al (2009)^107^	4511; Portugal; CS	7‐9 y	Parent report	BMI z (IOTF); BF%	✓ NEG (only in boys when analyzed separately)				7
Peach et al (2015)^108^	1364; USA; CS	11‐13 y	Self‐report	BMI	✓ NEG (boys Only)				7
Pesonen et al (2009)^109^	289; USA; CS	8(±0.3) y	Accelerometer (wrist)	BMI		NS (%)			3
Pileggi et al (2013)^110^	542; Italy; CS	9.9(± 0.4) ys	Parent report	BMI z (SIEDP)	✓ NEG				8
Prats‐Puig et al (2013)^111^	297; Spain; CS	5‐9 y	Self‐report	BMI z; WC; visceral fat	✓ NEG				6
Pryor et al (2015)^112^	1552, Canada, longitudinal	6‐12 (from 2.5‐5)	Parent report	BMI z (IOTF)	✓ NEG				4
Quach et al (2016)^115^	3631, Australia, LT	8‐9 (followed from 4 to 5)	Parent report	BMI z (CDC)				✓ POS (BT and timing cats)	7
Ramos and Barros (2007)^116^	2161; Portugal; CS	13 y	Self‐report	BMI z (CDC/IOTF)	✓ NEG (Boys Only)				7
Reilly et al (2005)^117^	7758; UK; LT	7 y (from 2.5)	Self‐report	BMI z	✓ NEG				6
Rosi et al (2017)^118^	690, Italy, CS	9‐11	Self‐report	BMI z (WHO)	✓ NEG			NS	3
Rudnicka et al (2017)^119^	4525, UK, CS	9‐10	Self‐report	BMI; bioelectrical impedance	✓ NEG				6
Santiago et al (2013)^120^	2814; Spain; CS	6‐12 y	Self‐report	BMI	✓ NEG (boys only)				4
Scharf and DeBoer (2015)^121^	7000; USA; CS and LT	5 y (from 4)	Parent report	BMI z (CDC); BMI tradectories	✓ NEG (LT only)			✓ POS (BT) NEG (WT)	4
Sekine et al (2002)^122^	8274; Japan; CS	6‐7 y	Parent report	BMI z (WHO)	✓ NEG			✓ POS (BT) NS (WT)	8
Shah et al (2013)^123^	200; India; CS	10‐12 y	self‐report	BMI	NS				4
Silva et al (2011)^124^	304; USA; CS (longitudinal out of age group)	6‐11 y	PSG (home)	BMI	NS				7
Stone et al (2013)^125^	856; Canada; CS	10‐12 y	Parent report	BMI z	✓ NEG				4
Sugimori et al (2004)^126^	8170; Japan; LT	6 y (from 3)	Parent report	BMI	✓ NEG (boys only)			✓ NEG (BT: girls only)	4
Suglia et al (2013)^127^	1589; USA; CS	5 y	Parent report	BMI z (CDC)	✓ NEG				3
Sun et al (2009)^128^	5753; Japan; CS	12‐13 y	Self‐report	BMI z	✓ NEG (girls only)			NS (BT; WT)	8
Taveras et al (2014)^129^	1046; USA; LT	7 yrs (from 1)	Parent report	BMI z, fat mass (DEXA); waist/hip ratio (at age 7)	✓ NEG				7
Thasanasuwan et al (2016)^130^	1345, Malaysia, Indonesia, Vietnam, and Thailand, CS	7‐12	Self‐report	BMI z (WHO)	✓ NEG				6
Thivel et al (2015)^131^	236; France; CS	6‐10 y	Parent report	BMI z; Fat Mas%				✓ POS (SMP)	3
Tovaret al (2012)^132^	401; USA; CS	6‐11 y	Parent report	BMI z (CDC)	✓ POS (OW NS OB)				7
Tuyet et al (2017)^133^	559, Vietnam, CS	6‐11	Parent report	BMI z (IOTF)	✓ NEG				4
Von Kries et al (2002)^134^	6645; Germany; CS	5‐6 y	Parent report	BMI z; Body fat mass (BIA)	✓ NEG				7
Wang et al (2016)^135^	16 028; China; LT	5 (from 3)	Parent report	BMI	✓ NEG				6
Wang et al (2017)^136^	5518; China; CS	9‐12	Self‐report	BMI z (WHO); BF%	✓ NEG		NS	✓ POS (BT)	9
Wells et al (2008)^137^	4452; Brazil; CS	10‐12 y	Self‐report	BMI, Skinfolds	✓ NEG				9
Wijnhoven et al (2015)^138^	15 643, Bulgaria, the Czech Republic, Lithuania, Portugal and Sweden, CS	6‐9	Parent report	BMI z (WHO)	✓ NS (all) NEG (Sig Portugal and Sweden)				7
Williams et al (2013)^139^	1215; New Zealand; LT	7 y (from 3)	Parent report	BMI z	✓ NEG				5
Wong et al (2013)^140^	333; USA; CS	9‐12 y	Accelerometry	BMI z	✓ NEG				7
Zhang et al (2016)^141^	3766, China, CS	7‐12	Self‐report	BMI z (WGOC)	✓ NEG				6

Abbreviations: Key: **✓,** significant association reported; BMI, body mass index; NEG, negative association report; POS, positive association report; NS, no significant association report; BT, bed time; WT, wake time; SWP, sleep wake pattern; SMP, sleep midpoint; CS, cross sectional; LT, longitudinal; %, sleep efficiency percentage; LWE, long wake episodes; IOTF, International Obesity Task Force 113; CDC, Centers for Disease Control 114; OW, overweight; OB, obese.

a Expanded notes on findings can be found in Table S3.

The included studies examined sleep across four dimensions (duration, timing, efficiency, and quality). Sleep duration was the most frequent dimension analyzed, with all but nine of the 112 studies reviewed assessing sleep duration. Of these 103 studies: 73 reported solely on the association between children's sleep duration and their weight status, 22 assessed duration and one other dimension, and seven assessed at least another two. Of the nine articles not reporting sleep duration, six solely reported on sleep timing factors, two on sleep quality, and one on sleep efficiency.

### 3.1 Sleep duration

Overall, there was strong evidence in support of an inverse association between primary school‐aged children's sleep duration and measured weight status. Of the 103 articles reporting on sleep duration: 86 (83%) reported a significant negative association between duration of sleep and measured weight status, where shorter sleep durations were linked with poorer weight status measures. In contrast, one study^132^ reported overweight and obesity to be higher among longer sleepers (>10 hours/night); while another study indicated mixed results, where girls with short sleep displayed higher weight status and boys with short sleep displayed lower weight status measures.^47^ Only 15 articles (14%) found no significant association between children's sleep duration and measured weight status.

Comparing measurement methods, sleep duration was mostly assessed subjectively through a calculation from self/proxy report bed (or sleep onset) and wake times, or self/proxy report duration (ie, “How many hours does your child usually sleep per day?”) (n = 84); with limited use of objectively assessed time spent asleep as determined through polysomnography (PSG) or accelerometry scoring of activity (n = 19). However, results were relatively similar across measurement methods, with a similar proportion of articles reporting an inverse association between longer sleep duration and healthier weight status as assessed via subjectively reported sleep duration (71/84; 85%), compared with objectively measured duration (16/19; 84%).

The longitudinal relationship between children's sleep duration and weight status outcomes was investigated by 22 studies, of which a significant negative association between shorter sleep durations and poorer weight status outcomes over time was reported across all but three.^74^, ^75^, ^101^ These three studies did however report significant cross‐sectional associations. While Hiscock et al^74^ reported a significant association between short sleep and higher weight status among children aged 6‐7 years, cross sectionally, initial sleep at 4 years did not significantly predict later weight status at age seven. Miller 101 and Hjorth et al 75 reported similar results.

There was also some evidence for gender patterning: with nine of the 103 reviewed articles reporting on sleep duration indicating the association between sleep duration and Ow/Ob as significant among boys only 62, 88, 89, 104, 107, 108, 116, 120, 126; while three reported it as significant among girls only^44^, ^77^, ^128^; and one reported mixed results across genders, as mentioned above.^47^

### 3.2 Sleep timing

Out of 112 reviewed articles, there were only 24 articles that reported on an association between the timing of children's sleep and their weight status (Table 2). Assessed components of sleep timing across these studies included either the timing of going to bed (bed time = BT) (n = 19), the time of waking (wake time = WT) (n = 10), sleep midpoint (n = 3), or sleep‐wake cycles (n = 4). Fifteen out of the 24 found a significant association between a timing componentry of sleep and Ow/Ob among children.

**Table 2:** Findings from sleep timing articles

Article	Timing factor/s			Measure of timing
	Bed time	Wake time	Other	
Alqaderi et al (2017)^34^	✓ Pos (LT)			Self‐report
Amigo et al (2014)^36^	✓ Pos			Self‐report
Anderson et al (2017)^37^	✓ Neg (consistent bed‐time at age 3; compared with inconsistent)			Parent report
Berentzen et al (2014)^44^			NS (sleep‐wake pattern)	Self‐report
Busto‐Zapico et al (2014)^45^	✓ Pos			Parent report
Cassimos et al (2011)^51^	NS			Self‐report
Combs et al (2016)^55^	✓ Pos (weekday)	NS (weekday)		Parent report
Ekstedt et al (2013)^63^	NS	NS		Accelerometry
Firouzi et al (2013)^67^	NS	NS		Parent report
Harrex et al (2017)^72^			NS (sleep‐wake pattern)	Accelerometry
Kelly et al (2015)^80^	✓ NEG (nonregular BT) POS (BT > 9 pm; LT)			Parent report
Khan et al (2017)^81^	✓ Pos (weekday)			Parent report
Lumeng et al (2007)^94^	✓ Pos	NS		Parent report
Martoni et al (2016)^99^			✓ Pos (sleep midpoint)	Accelerometry
McNeil et al (2015)^22^	NS	NS	NS (Sleep midpoint)	Accelerometry
Ochiai et al (2012)^104^	NS	NS		Parent report
Quach et al (2016)^115^	✓ Pos (LT: as dichotomous categorization of LTB)		✓ Pos (LT: wave 2 only) (sleep‐wake pattern)	Parent report
Rosi et al (2017)^118^			NS (sleep‐wake pattern)	Self‐reported
Scharf and DeBoer (2015)^121^	✓ Pos (CS and LT)	✓ Neg (CS only)		Parent report
Sekine et al (2002)^122^	✓ Pos	NS		Parent report
Sugimori et al (2004)^126^	✓ Pos (Girls Only; LT)	NS		Parent report
Sun et al (2009)^128^	NS	NS		Self‐report
Thivel et al (2015)^131^			✓ Pos (sleep midpoint)	Parent report
Wang et al (2017)^136^	✓ Pos			Self‐report

Abbreviations: Key: **✓** = significant association reported; NS = no significant association with weight status; Pos = positive association (later BT OR WT ➔ higher weight status); Neg = negative association (earlier BT OR WT **➔** higher weight status); BT = Bed Time; WT = Wake Time; LTB = late to bed; CS = cross‐sectional association; LT = longitudinal association.

A trend was found supporting a positive association for the relationships between later bed‐timing/sleep onset and Ow/Ob among children. Of the 19 articles reporting on children's bedtime (BT), 12 indicate later bed‐times/sleep onset were linked with significantly poorer weight status (though one of these was significant among girls only; and two for weekday BT only). These findings were consistent across both self‐reported and parent‐reported BT, cross sectionally and longitudinally, with 10 of 12 studies with parent‐reported BT and three of five studies with self‐reported BT reporting significant links between later or inconsistent bed times and increased overweight/obesity. The remaining six out of the 19 articles (32%) reporting on BT and Ow/Ob among children, found no association between BT and children's weight status, two of which were the only studies with accelerometry determined BT.^22^, ^63^

Results were mostly consistent when assessing the impact of wake time (WT), regardless of being assessed from parent report, self‐report or measured via accelerometry. Of the total 112 reviewed articles, only 10 papers examined the association between WT and weight status among children, of which only one reported a significant association. Scharf and DeBoe^121^ reported that children aged five waking before 6:30 am were significantly more likely to be affected by obesity (OR = 1.23, 95%CI 1.01‐15.51, *P* < .05), although this association was not observed amongst their longitudinal sample comparing WT at 4 years of age and weight status at 5 years of age.

There was less examination across the other aspects of sleep timing including sleep midpoint (the half‐way point/time between sleep onset and wake time) and sleep‐wake patterns. Only three studies reported on sleep midpoint, of which two reported significant positive associations. Both Thivel et al^131^ and Martoni et al)^99^ indicated that measures of overweight and obesity were significantly higher among children who demonstrated a later sleep midpoint.

The final aspect of sleep assessed was sleep‐wake patterns, determined according to consideration of combined dichotomized categorization of BT and WT, creating the sleep timing labels of early to bed/early to rise; early to bed/late to rise; late to bed/early to rise; and late to bed/late to rise. Only one of the four studies assessing this factor found a significant association reporting higher BMI z‐scores among late to bed/early to rise profiles compared with early to bed/early to rise sleepers, although this was only significant among 6‐7 year olds and not 8‐9 year olds. 115

Of the total 112 reviewed studies and 24 timing studies, only four^22^, ^63^, ^72^, ^99^ measured sleep timing objectively via accelerometry. One of these assessed sleep‐wake timing,^72^ which supported the general nonsignificance consensus. Two of the studies^22^, ^67^ reported on the association between BT and WT with children's BMI z‐scores, both reporting nonsignificant results. While these findings were consistent with those from both parent reported and self‐reported WT, these contrasted the general consensus found for later self‐/parent‐reported BT and increased weight status among children.

McNeil et al^22^ also assessed sleeping midpoint, as did and Martoni et al .^99^ These studies had converse findings with Martoni reporting a significant positive association, which contrasted the nonsignificant findings reported by McNeil. However, the only other study reporting on sleep midpoint, findings from Thivel et al found a positive association between parent reported later sleep midpoints and higher percentage fat mas among children, further supporting the positive association.^131^

### 3.3 Sleep efficiency

Twelve studies examined an aspect sleep efficiency; analyzing either sleep latency (the efficiency of initiating sleep), maintaining sleep (wake episodes), or as a time percentage variable.

The percentage of time spent asleep, calculated as the time between sleep onset and waking, was assessed by seven of the 12 papers. These seven studies used accelerometry to objectively measure the efficiency, with four reporting a significant negative association between higher sleep efficiency scores and poorer anthropometric outcomes among children, while three found no significant association (Table 3).

**Table 3:** Findings from sleep efficiency articles

Article	Classification of efficiency measure				Measure method
	Efficiency %	Sleep onset delay	Night awakenings	Measure	
Arora and Taheri (2015)^39^	NS			The percent of time between sleep onset and wake time spent asleep	Accelerometry (wrist)
Bagley and El‐Sheikh (2013)^40^	✓ Neg			The percent of time between sleep onset and wake time spent asleep	Accelerometry (wrist)
Bagley and El‐Sheikh (2014)^20^	✓ Neg		✓ NS (sleep activity) Pos (long wake episodes)	The percent of time between sleep onset and wake time spent asleep; long sleep wake episodes; sleep activity	Accelerometry (wrist)
Berentzen et al (2014)^44^			NS	Frequency/length of night awakenings	Self‐report
Ekstedt et al (2013)^63^	NS			The percent of time between sleep onset and wake time spent asleep	Accelerometry (wrist)
El‐Sheikh et al (2007)^64^	✓ Neg			The percent of time between sleep onset and wake time spent asleep	Accelerometry (wrist)
Fernandez‐Mendoza et al (2014)^65^		✓ (combined) NS		“has trouble falling asleep” or “wakes up often in the night”	Parent report
Firouzi et al (2013)^67^		NS		Sleep onset delay	Parent report
Liu et al (2011)^92^		NS	✓ Pos	One item on trouble falling asleep; one item on waking up during night; One item restless at night	Parent report
Lumeng et al (2007)^94^			NS	Night waking problems	Parent report
McNeil et al (2015)^22^	✓ Neg			The percent of time between sleep onset and wake time spent asleep	Accelerometry (waist)
Pesonen et al (2009)^109^	NS			Actual sleep time divided by the time in bed	Accelerometry (wrist)

Abbreviations: Key: **✓** = significant association reported; NS = no significant association with weight status; Pos = positive association (higher % or high problems or high awakenings leads to higher weight status); Neg = negative association (lower % or low problems or low awakenings leads to higher weight status); CS = cross‐sectional analysis.

Two further aspects of sleep efficiency were identified across six of the 12 papers; sleep latency issues (delay/difficulty initiating sleep), and night awakenings (problems with or frequency/duration of wake episodes). Sleep initiation was assessed by two studies,^67^, ^92^ night awakenings by four,^20^, ^44^, ^92^, ^94^ and one paper combined the two factors as a single item.^65^ No significant association between sleep onset delays and children's weight status was reported (both parent reported). Only night awakenings were found to be a significant predictor across two studies. Liu et al reported significantly higher proportion of Ow/Ob children had issues with sleep maintenance, reporting more night awakening problems (parent reported) compared with normal weight children (*P* < .05),^92^ while Bagley and El‐Sheikh reported that the number of wake episodes (assessed via accelerometry) was only significant if these episodes were ≥ 5 minutes each.^20^

### 3.4 Sleep quality

Of all 112 studies reviewed, 13 analyzed the association between quality of sleep and children's weight status (Table 4). All 13 studies collected subjective measures of sleep quality, ranging from a single item question on whether sleep problems were present, to the sum of scores across 10‐33 items relating to multiple aspects of sleep quality. The measurement method across the studies was different for all 13 studies. While the Child Sleep Health Questionnaire was used in four studies, each used different adaptations or modified version. Results were mixed, with four of six studies with self‐reported sleep quality and three of seven studies with parent‐reported sleep quality reporting no significant association between children's anthropometric measurements and the reported quality of sleep. The remaining six studies indicate a negative association, where higher levels of reported sleep problems lead to higher risk of Ow/Ob.

**Table 4:** Findings from sleep quality articles

Article	Measure	Measure method	Association
Alamian et al (2016)^33^	Infant sleep problems: score of multiple sleep measure, according to three classifications	Parent report	✓ NEG (LT)
Berentzen et al (2014)^44^	Difficulty getting up, feeling rested, daytime sleepiness	Self‐report	NS
El‐Sheikh et al (2014)^21^	School Sleep Habits Survey (10 item sleep wake problem scale)	Self‐report	✓ NEG (LT, girls only)
Ferrari et al (2017)^66^	The Diet and Lifestyle Questionnaire (how well slept)	Self‐report	NS
Firouzi et al (2013)^67^	CSHQ: parent report total sleep disorder score (34 items)	Parent report	✓ NEG
García‐Hermoso et al (2017)^68^	Sleep Self‐Report (SSR), Spanish version (19 items)	Self‐report	✓ NEG (girls only)
Hiscock et al (2011)^74^	Parent report: on sleep problem (single question‐none/mild); sleep problem (mod/severe)	Parent report	NS
Hjorth et al (2014)^75^	CSHQ: parent report sleep disturbances (33 item)	Parent report	NS (CS and LT)
Jing Jing et al (2017)^78^	Likert scale (very good, good, fair, poor, very poor)	Self‐reported	NS
Khan et al (2017)^81^	Likert scale (child snores, if they ever wake up unrefreshed in the morning and if they were sleepy during the daytime), dichotomized into good vs poor	Parent report	✓ NEG
Liu et al (2011)^92^	Sleep Behavior Questionnaire: total sleep behavior score (11 items on sleep problems)	Parent report	✓NEG
Lumeng et al (2007)^94^	CSHQ: parent report sleep disturbances General sleep problem score (27 items)	Parent report	NS (CS and LT)
Wang et al (2017)^136^	CSHQ: self‐report total sleep disorder score (21 items)	Self‐report	NS

Abbreviations: CSHQ = Child Sleep Health Questionnaire; ✓ = significant association reported; NS = no significant association with weight status; NEG = negative association with weight status (better sleep quality reduces weight status); CS = cross‐sectional association; LT = longitudinal association.

Results also varied longitudinally, with two of the four studies assessing the impact of sleep quality on weight status over time reported results to be non‐significant. Hiscock et al^74^ and Lumeng et al^94^ found no significant association across their time points. Furthermore, out of the two studies indicating lower sleep quality to be associated with poorer weight status,^21^, ^33^ one found the association as significant among girls only.^21^ El‐Sheikh et al,^21^ found that those who reported higher sleep problems at T1 (9 years old) had higher BMI scores at T3 (11 years old), although this was only significant among girls.

## 4 DISCUSSION

This systematic review highlights a strong importance on assessing sleep as a behavioral factor associated with increased risk of Ow/Ob among primary school aged children across multiple studies set in multiple countries. Out of the 112 reviewed studies, 98 reported a significant association with a dimension of sleep and increased weight status. Supporting findings from previous reviews, the current review found 86 of 103 (83%) studies found a significant negative association between sleep duration and overweight and obesity among children aged between five to thirteen years old. As previously reported,^9^, ^10^, ^11^, ^13^, ^14^ this association has been demonstrated cross sectionally as well as longitudinally, using both subjectively and objectively measured duration of children's sleep.

Of particular interest to the current review, is the in‐depth investigation of the association between other dimensions of sleep (namely sleep efficiency, quality, and the timing of both bed and wake times) and Ow/Ob among primary school aged children. Beyond those of previous reviews, the current article highlights that outside the heavily researched influence of the duration of sleep, aspects of the outlined sleep dimensions potentially have significant and independent influence in the sleep‐obesity association.

Compared with sleep duration, we found less evidence of studies examining other dimensions of sleep, such as quality, efficiency and bed/wake times, in attempts to understand the sleep‐obesity relationship. Where studies exist, there is evidence of associations across these additional dimensions on the sleep‐obesity nexus, particularly supporting a positive association with later bed timing and increased weight status. Less consistent findings were found across studies exploring components of sleep efficiency and sleep quality; however, the variations in these findings appear to relate to the inconsistencies of outlined definitions and measurement tools utilized. From previous definitions, sleep efficiency relates to the ease and continuity of initiating and maintaining sleep, while quality surrounds either measurement of specific sleep wave or perceived satisfaction/perceived problems with sleep.^15^, ^16^ The term sleep quality appeared to be used as an interchangeable/overreaching term for many of these aspect of sleep, some which appear more relevant to sleep maintenance/efficiency. Each of the reviewed studies also utilized a unique variation to obtain perceived quality or an overall sleep quality score. The variation in measurement approach and definition of these dimensions creates difficulties in comparing results across the literature. However, despite these discrepancies, this review indicates the importance of considering the influence of different dimensions when examining the sleep‐obesity nexus.

This review identified several timing factors that can be measured, including bedtime, wake time, sleep midpoint, and sleep timing patterns. Across the reviewed articles bedtime appears to be more influential than wake time, with a much stronger evidence for the association of later bedtimes and increased weight status among school children,^36^, ^45^, ^94^, ^121^, ^122^ than that of later wake times.^121^ Considering the target population, this could mostly be due to the commonality of the daily school routine, acting as a regulator on morning wake times/schedules, and more individual variability on bedtime routines.^142^

One of the other two measures of sleep timing minimally outlined included sleep midpoint, which used as a single marker (the midpoint) to assess the timing of sleep, taking duration completely out of the equation. 22, 99, 131 The second measure was sleep wake cycles, almost a combined sleep timing/duration variable, categorising children as late to bed/wake early (shortest duration), early to bed/wake early or late to bed/wake late (mid duration), and early to bed/wake late (long duration).^44^, ^72^, ^115^, ^118^ While results from the reviewed studies potentially suggest a stronger importance of sleep midpoint than sleep‐wake patterns, it is still unclear how reliable/useful these measures of sleep timing are when analyzing the sleep‐obesity association, due to the limited number of studies with these measures of timing and the discrepancies across results. It could be hypothesized that compared with WT and sleep‐wake patterns, bedtime and sleep midpoint might be more significant factors in the sleep‐obesity nexus, independently from sleep duration,^18^, ^45^ due to potential night‐time behaviors associated with delayed sleep.

With limited articles reporting on the longitudinal association between sleep timing and children's weight status, the etiology of the association is hard to determine. However, one proposed mechanism suggests children with later bedtimes have higher TV viewing/screen time and poorer snacking/dietary habits.^36^, ^45^, ^121^, ^131^ Busto‐Zapico et al indicate children with later bedtimes are more likely to have a higher BMI, especially when they use the time that they should be sleeping engaged in sedentary leisure activities (watching TV, computer games, etc.),^45^ while Thivel et al found children with later sleep midpoint, compared with normal sleepers, had poorer eating habits measured by number of cumulated eating risk factors (eg, snacking and sweetened beverage consumption).^131^ It could be proposed that later sleep timing not only contribute to a disrupted energy balance directly through reduced restorative sleep (reduced duration due to consistent WT) but also by generating increased time available for these additional obesity risk factor behaviors. Further evidence is required to unpack these associations.

This review found contrasting evidence across both night awakenings and objectively measured efficiency percentage (percentage of time spent asleep during the period between sleep onset and waking).^20^, ^22^, ^40^, ^64^, ^92^ While most subjectively assessed awakenings were reported as non‐significant, Bagley and El‐Sheikh,^20^ the only study with objectively measured night awakenings (via accelerometry), reported that although the number of wake episodes alone was nonsignificant with BMI, however the number of long wake episodes (those longer than 5 minutes) was associated with a significant increase in BMI among children. Contrasting evidence was also reported among sleep quality studies, with significant associations of poorer sleep quality and increased obesity risk reported among less than half of those exploring self or parent determined sleep quality. There was a lack of consistency in measurement approach across all reviewed studies reporting on children's sleep quality and efficiency. While four of the sleep quality studies used the same tool (the Child Sleep Habits Questionnaire [CSHQ]), minor alterations and differing questionnaire items were included across these. The variation in measurement approach and definition of these dimensions could be impacting findings. There is also a risk of error associated with potential recall bias from self‐report among young children, or reporter bias through proxy report by parents.^143^ The use of measures such as PSG or accelerometry might provide more objective assessment of these dimensions and should be used where possible. However, self‐/proxy‐reported measures are practical and cost‐effective and are deemed more suitable in population‐based research.^143^, ^144^ Pragmatic considerations on research methodology therefore call for enhanced clarity around definitions along with development of further psychometrically tested measurement tools to examine these dimensions among the target population.

Despite the variations, understanding the impact of both sleep efficiency and quality of sleep on the sleep‐obesity association should be of interest of future research as some research has indicated this association could be independent from the duration of sleep.^67^, ^145^ Delayed sleep initiation and poor sleep maintenance not only potentially reduce overall sleep duration but can also, as with delayed sleep timing, present available time for snacking. As the length of wake episodes is reportedly more influential on weight status outcomes, rather than simply the number of episodes,^20^ it could be suggested that these periods provide opportunity for snacking. Higher caloric intake has been recorded among clinical samples of adults with sleep related eating disorders in America, where higher wake‐episode frequencies were associated with increased snacking after initial sleep onset and overall daily calorie consumption.^146^, ^147^ However, research on this mechanism is limited, particularly among children. Future research would benefit from exploring how time in wake episodes is spent and the impact this might have on the sleep‐obesity nexus.

Furthermore, factors such as reduced sleep duration, later timing and poor sleep maintenance could impact sleep quality, altering hormone levels due to disrupted structure and timing of specific sleep wave cycles.^148^ Optimal sleep quality usually involves around four cycles of sleep, through slow wave sleep to REM sleep, which is important for endocrine and metabolic regulation. Kim et al review how impeded sleep quality and disruptions to these sleep cycles has been linked with insulin insensitivity, dysregulation of appetite hormones (leptin and ghrelin), decreased melatonin and metabolic disruption among adults.^148^ Sleep curtailment studies among adults have shown altered hormones such as leptin could negatively impact weight status outcomes through altered hunger signals, resulting in higher caloric consumptions.^149^ Additionally, disruptions to melatonin have been associated with increased daytime sleepiness among clinical samples of adults and children,^150^, ^151^, ^152^ which has been shown to influence physical activity levels and increased obesity risk among samples of adults in America^153^, ^154^ and school‐aged children in Japan.^155^

In agreeance with a recent review that evaluated the association between sleep quality and obesity among children and young adults,^145^ more research is required to explore how these factors impact each other and the sleep‐obesity relationship, along with the need for clarity on defining and assessing these dimensions.

### 4.1 Implications of the study

This review highlights the importance of assessing and understanding the impact of the multiple dimensions of sleep when investigating the association between sleep and weight status among children. The results propose that children's bedtimes, quality of sleep, and sleep efficiency could be equally as influential on the sleep‐obesity association (among children) as the prolifically researched dimension of sleep duration. While the consensus of this association appears stronger for the duration of sleep, this review also highlighted the importance of bed times/sleep patterns in the sleep‐obesity nexus. Less congruent findings were found for sleep quality and efficiency measures; however, this could be due to the comparatively limited studies investigating these dimensions when assessing adequate/inadequate sleep among children. The inconsistencies of measurement methods and definitions across the literature also create some discrepancies in results. To better understand the role of these dimensions on the sleep‐obesity association, future research would benefit from clarity on definitions across the different dimensions along with the use of valid and reliable tools.

Furthermore, due to the scarce longitudinal data across the reviewed studies (outside of sleep duration), the etiology of the association is hard to determine. To better determine the causality on the association between children's weight status and the multiple sleep dimensions, changes in these variables need to be examined over time among population samples, as well as exploring the impact of potential covariates (ie, screen time, diet, and physical activity).

Whilst further research is needed, there is consensus in the literature around the importance of the timing of sleep and not only sleep duration on the sleep‐obesity nexus. Clinicians should therefore consider all dimensions of sleep but particularly children's sleep timing and duration in their practice.

### 4.2 Strength and Limitations

To the authors' knowledge, this is the first systematic review that has explored and compared several dimension of sleep in relation to the sleep‐obesity association among children. A strength of the current study was the large number of studies systematically reviewed in order to broadly cover several active dimensions in the sleep‐obesity association among children. However, while the inclusion criterion of objectively measured weight status may have created a limitation on the number of included studies (potentially limiting a greater representation of each of the sleep dimensions), this criterion minimized the risk of variability due to measurement of the outcome variable.

Furthermore, focusing on free‐living populations and the defined age group allowed for a clearer indication of the association on children, as opposed to adolescents, as sleep needs and behaviors can be affected/altered among some clinical populations and across age groups.

While the large spread of studies provides a greater exploration of the multiple dimensions in the sleep‐obesity association among children, it also creates some restrictions. The multiple measurement and definition discrepancies within the dimensions complicated and limited comparisons of results across studies and dimensions, hence why no meta‐analysis was conducted. This review was also limited in the capacity to compare findings across potentially linked/covariate factors (that might influence the sufficiency of sleep attained (across the dimensions) and influence the sleep‐obesity association. For example, one factor potentially relevant to the sleep‐obesity relationship not detailed in this review was the impact of tanner stage/maturation. This was due to the limited number of studies^22^, ^50^, ^71^, ^90^, ^97^, ^98^, ^125^, ^137^ reporting/controlling for this covariate making it impossible to include this in comparisons of papers.

## 5 CONCLUSIONS

Similar to previous reviews, this study found strong evidence for a negative association between sleep duration and overweight and obesity among primary school‐aged children. However, inconsistencies in both measurement method and definition of sleep quality, sleep efficiency, and sleep timing limited the conclusions that could be drawn for these dimensions. To better understand the role of these dimensions on the sleep‐obesity association, future research would benefit from clarity on definitions across the different dimensions along with the use of valid and reliable tools. Furthermore, a combined approach should be incorporated in future research, to investigate these dimensions simultaneously and longitudinally so that the potential influence of the multiple dimensions of sleep on children's weight status can be analyzed overtime and as sleep needs change.

## CONFLICT OF INTEREST

The authors have no conflicts to disclose.

## AUTHOR CONTRIBUTIONS

B.M. and C.S. were responsible for the literature review and data extraction. B.M. was responsible for the data analysis and drafting of paper. S.A., C.S., and E.T. provided critical review for the final manuscript.

## Supporting information


**Appendix** Supporting Figures and Tables
