# Supplementary material for: Sleep and obesity among children: A systematic review of multiple sleep dimensions
Source: Pediatr Obes. 2020 Feb 18;15(4):e12619. doi: 10.1111/ijpo.12619 (PMC7154640; doi:10.1111/ijpo.12619)
Supplement: Supplementary file 1 — Appendix Supporting Figures and Tables [file IJPO-15-e12619-s001.docx]

# Sleep and obesity among children: A systematic review of multiple sleep dimensions

Authors: Bridget Morrissey (Hons)^1,2^, Elsie Taveras (PhD)^3,4^, Steven Allender (PhD)^1,2^, Claudia Strugnell (PhD)^1^

1. Deakin University, Geelong, Australia, Global Obesity Centre, Centre for Population Health Research
2. Deakin University, Geelong Australia, School of Health and Social Development
3. Division of General Academic Pediatrics, Department of Pediatrics, Massachusetts General Hospital for Children, Boston
4. Department of Nutrition, Harvard T.H. Chan School of Public Health, Boston, Massachusetts, USA

| Table S1: Search Strategy# | |
| --- | --- |
| Sleep Behaviours | (Sleep* OR "sleep depriv*" OR "sleep duration*" OR "sleep behavio#r*" OR "sufficient sleep*" OR "insufficient sleep*" OR "short sleep*" OR "sleep tim*" OR "bed tim*" OR bedtim* OR "time in bed" OR "sleep hygiene" OR "sleep efficiency") |
| **AND** | |
| Obesity/weight status | (overweight or over-weight or "weight gain" or "weight status" or bmi or "body mass index" or "body mass" or bmiz or "standardi?ed bmi" or obes* or "body weight" or "body size") |
| **AND** | |
| Child/Adolescent | (child* or adolesc* or p#ediatr* or "school child*" or schoolchild* or youth* or teen* or "young person" or "young people" or "young adult*" or boy* or girl* or minor* or juvenile*) |
| #Terms based on previous reviews ^1-6^ | |

| Table S2: Data Extraction Template | | | | | | | | | | | | | | | | | | | | | | | | | | |
| --- | --- | --- | --- | --- | --- | --- | --- | --- | --- | --- | --- | --- | --- | --- | --- | --- | --- | --- | --- | --- | --- | --- | --- | --- | --- | --- |
| Author | Title | Study Design | Study Sample Name | Country | Year of data | Sample Size | Response Rate | Gender | Age Group | Measure of Weight Status | Measure of Sleep Dimensions | Validated yes/ no | Confounding Variables Addressed | If assessed:  NS or Sig Association | | | | Pattern and Association | Quality Score | 1. Representativeness of sample | 2.Sample size | 3. Non-respondents | 4. comparable groups | 5. Assessment of sleep | 6. Assessment of exposure | 7. Adequate follow-up/ Statistical test |
|  |  |  |  |  |  |  |  |  |  |  |  |  |  | Sleep Duration | Sleep Efficiency | Sleep Quality | Sleep Timing |  |  |  |  |  |  |  |  |  |
|  |  |  |  |  |  |  |  |  |  |  |  |  |  |  |  |  |  |  |  |  |  |  |  |  |  |  |

| Table S3: Study Findings Explained | |
| --- | --- |
| **First Author (year)** | **Pattern and association** |
| Agras et al. (2004) ^7^ | Sleep duration at 3 to 4 years was negatively associated with weight at 9, with an average of 30 minutes less sleep among those who became overweight compared with those who would remain normal weight. |
| Alamian et al. (2016) ^8^ | After adjusting for all covariates, infant sleep problems were found to be associated with an increased risk of being overweight in 6th Grade using either Zuckerman et al.'s (OR = 1.68; 95% CI: 1.11–2.55) or Richman's definition (OR = 1.76; 95% CI: 1.05–2.97), but not using Lozoffet al.'s definition (OR=1.07; 95% CI: 0.66–1.74) |
| Alqaderi et al. (2017) ^9^ | Statistically significant longitudinal association between increases in WC with later bedtime (P<0.05) |
| Altenburg et al. (2013) ^10^ | Negative association between sleep duration and BMI/WCc for participants sleeping less than 10 hours/night. i.e 7 hours or less instead of 10 hours per night increased mean BMI by 0.98 kg/m2 and mean WC by 2.35 cm, p<0.05 |
| Amigo et al. (2014) ^11^ | Bed times (r=0.15,p<.01) and sleep duration (r=-.15,p<.05) were correlated with BMI |
| Anderson et al. (2017) | children with inconsistent bedtimes at age 3 were more likely (OR (95% CI) = 2.18 (1.70–2.79)) than children who always had a regular bedtime to be obese at age 11 |
| Anujuo et al. (2016) | There was no significant relationship between sleep duration and mean BP and raised BP, and overweight in all groups |
| Arora and Taheri (2015) ^12^ | A significant negative association between average weekday, weekend and overall sleep duration (estimated by actigraphy) and BMI z-score was observed β = − 0.36, P = 0.001; β = − 0.11, P40.05; and β = − 0.36, P ≤ 0.001, respectively. Sleep efficiency was not, however, significantly associated with BMI z-score after adjustment. |
| Bagley and El-Sheikh (2013) ^13^ | Shorter sleep duration predicted greater BMI z-score, β = -0.01, t(222)= -2.60,p<.01.; Sleep efficiency predicted BMI z-score at the trend level only, b=-0.02, t(222)= -1.37, p<.10 |
| Bagley and El-Sheikh (2014) ^14^ | Significant negative relationship between BMI and Sleep minutes (β **=**-0.26, P<0.01), sleep efficiency (β **=**-0.18, P<0.05), no sig relationship with sleep activity and significant positive relationship with long wake episodes (0.17, P<0.01). |
| Barlett et al. (2012) ^15^ | Mediated path analyses showed Wave 2 sleep duration predicted Wave 3 BMI (β **=**-.03, P<0.03). Bivariate analyses found sig correlation between Wave 1 sleep and Wave 1 BMI ( r = -0.17, P<0.05); Wave I BMI and Wave 2 sleep (r = -0.18, P<0.01); Wave 1 sleep and Wave 3 BMI (r=-0.16, P<0.01); Wave 2 sleep and Wave 3 BMI (r = -0.20, P<0.01) |
| Bayer et al. (2009) ^16^ | As sleep duration increases, mean BMI z-score and KFA-z scores decreased. Estimated betta coefficients and 95% CI from Figure 2: 6 Years of age (β **=**-0.25, 95%CI (-0.4; -0.1); 7 Years of age (b= -0.20; 95% CI (-0.3; -0.1); 8 Years of age (b= -0.2; 95% CI (-0.35; -0.1); 9 Years of age (b=-0.3, 95% CI (-0.45; -0.15); 10 Years of age (b= -0.2, 95% CI -0.35; -0.8) |
| Bell and Zimmerman (2010) ^17^ | Children with short sleep duration at 0-4 years of age had higher odds of overweight/obesity at follow-up (OR 1.80; 95% CI 1.16, 2.80), NS for older Cohort after baseline BMI included in model. Low sleep at follow-up sig higher odds for overweight/obesity at follow-up among older cohort (OR 2.20, 95% CI: 1.79,2.69) NS for younger cohort |
| Berentzen et al. (2014) ^18^ | Odds of being overweight decreased for girls with a long time in bed by 56% [odds ratio (OR) = 0.44, 95% CI 0.24; 0.79] compared with girls with a medium time in bed; no association was found in boys. Non-significant associations with BMI and WC with sleep latency type (e.g. early to bed/early to rise), sleep quality, daytime outings, feeling rested, feeling sleepy for both boys and girls. |
| Busto-Zapico et al. (2014) ^19^ | BMI was significantly correlated with bedtime and sleep duration (p<0.01). The relationships between insufficient sleep and an increase in BMI appear to be mediated by sedentary leisure activities. A reduction in hours of sleep in the children predicted greater levels of BMI if these children used the time when they should have been sleeping to watch the television |
| Cameron et al. (2013) ^20^ | Significant negative associations were observed for average sleep duration and BMI among boys [β **=** -0.47 (0.09), p<0.001)] and girls [B -0.29 (0.09), P<0.001) and for WC for boys [β **=**-1.12(0.25), p<0.001] and girls [β **=** -0.69 (0.22), P<0.002] |
| Cao et al. (2015) ^21^ | Short sleep duration is associated with increased chances of obesity among girls, but decreased among 6- to 12-year-old boys. OR comparing <7hrs with >=9hrs was 0.60 (95 % CI: 0.55–0.66) for boys |
| Carrillo-Larco et al. (2014) ^22^ | Adjusted models found no significant association between short sleep duration and overweight or obesity compared with regular sleepers for both boys and girls separately. |
| Carter et al. (2011) ^23^ | Adjusted models showed that each additional hour of sleep at aged 3-5 was associated in -0.39 (-0.72, -0.06) BMI at age 7. An additional hour of sleep 3-5y reduced the odds of overweight of 0.39 (0.24, 0.63) at age 7. Each additional hour of sleep at age 3-5y reduced fat mass index by -0.48 (-0.86; -0.10) |
| Casazza et al. (2011) ^24^ | No significant association between adiposity measures (total fat, % fat or BMI) and sleep duration |
| Cassimos et al. (2011) ^25^ | No significant difference in mean hours of sleep duration between normal weight (8.75 +/- 0.78 h) and overweight (8.72 +/- 0.66; or normal weight and obese (8.65 +/- 0.65); bed time NS difference across weight categories |
| Chahal et al. (2013) ^26^ | For every additional hour of sleep, the odds of being overweight at Grade 5 decreased (OR = 0.72 (95%CI: 061; 0.87) and for obesity (OR = 0.71 (95% CI: 0.54; 0.90) in adjusted models. |
| Chaput and Tremblay (2006) ^27^ | Compared to children reporting 12–13 h of sleep per day, the adjusted odds ratio for childhood overweight/obesity was 1.42 (95%CI: 1.09–1.98) for those with 10.5–11.5 h of sleep and 3.45 (2.61–4.67) for those with 8–10 h of sleep |
| Colley et al. (2012) ^28^ | Parent-reported sleep duration was not significantly correlated with BMI or WC; however, accelerometry measured sleep duration (hours/day) was significantly associated with BMI (-0.318, P<0.05) but not WC in the fully adjusted model. In models only adjusted for age and sex, parent-reported sleep (hours/day) and measured sleep was not significantly associated with BMI and WC. |
| Combs et al (2016) ^29^ | Among phase one 7-10 year olds; weekday increased sleep duration was significantly associated with decreased BMI percentiles (b=-0.005, p<0.0001), while weekend sleep duration was not; additionally Weekday bedtimes was positively associated with increased BMI percentiles (b=0.003, p<0.05), while wake times was not. |
| De Jong et al. (2012) ^30^ | Short sleep duration (≤ 10.25 hours/night) was associated with overweight in boys (OR = 4.96 (95% CI: 1.35-18.16) and girls (OR = 4.86 (95% CI: 1.59; 14.88) |
| Del Pozo-Cruz et al (2017) ^31^ | among the sub sample of 5-9 year olds, BMI was negatively associated with sleep duration (b=-0.0101, 95% CI -0.016 - -0.004, p<0.05) |
| Diethelm et al. (2011) ^32^ | Significantly higher FMI was evident among consistently short sleepers to age 7, alongside a higher odds for excess body fat at age 7 for the consistently short (OR = 2.4; 95% CI: 1.3, 4.6) and inconsistent (OR = 1.4, 95%CI: 0.7, 2.8) compared to the referent of consistently long sleepers. |
| Drescher et al. (2011). ^33^ | Significant negative association between total sleep-time and BMI (β **=** -0.002, (95% CI: -0.005, 0.000) Parent-reported total sleep time was negatively associated with BMI (r= -0.160, p<0.01). No significant relationship with PSG-recorded total sleep time or child reported total sleep time was observed. No significant association found with triceps skin fold. |
| Duncan et al. (2008) ^34^ | Weekday sleep duration was negatively associated with excess BF% compared to those that slept ≥ 12 hrs [≤ 10 hr OR = 7.03 (95%CI: 1.63-30.4), 10-10.9hr OR = 4.23 (95% CI: 1.13, 15.8), 11-11.9hr OR = 3.92 (95% CI: 1.07, 14.4). No significant association was observed for weekend association. |
| Duran & Haro (2016) ^35^ | Odd ratio for overweight/obesity when categorised as a short sleeper during the week (<10 hours) were significantly increased (crude OR 1.82; CI 95%: 1.29-2.56, adjusted model 2 OR 1.83; CI 95%: 1.30-2.59 and model 3 OR 1.85; CI 95%: 1.30-2.62). However looking at weekend sleep, no significant association was found. |
| Eisenmann et al. (2006) ^36^ | Boys aged 7.5-10.9y OR = 2.42 (95% CI: 1.14, 5.15) and Boys aged 11.0-13.9 (OR = 2.73 (95% CI: 1.53, 4.85) who slept ≤ 8hrs had a higher odds of overweight than those who slept ≥ 10hrs. No significant association for girls in logistic regression analyses. Bivariate analyses of mean WC showed significant differences in mean WC among boys and girls aged 7.5-10.9y who slept 8-9hrs compared to those who slept ≥ 10hrs, and for boys (only) aged 11-13.9y). |
| Ekstedt et al. (2013) ^37^ | A negative correction between total sleep time and BMI (r=-.10, P<0.01) was found. No significant association between sleep efficiency, sleep onset or sleep end (wake up) were found |
| El-Sheikh et al. (2007) ^38^ | Adjusted hierarchical regression analyses found significant associations between BMI and wake minutes (B =0.22, P<0.01), sleep minutes (B= -0.24, P<0.01), and sleep efficiency (-0.23, P<0.01). |
| El-Sheikh et al. (2014) ^39^ | Bivariate analyses found a correlation between BMI T1 and sleep minutes at T1 (r = -0.20, P<0.05), BMI T2 and sleep minutes T2 (r = -0.41, P<0.001), BMI T3 and sleep minutes T3 (r = -0.34, P<0.001). Longitudinal associations were found between BMI at T1 and sleep minutes at T2 (r=-0.38, P<0.01) and T3 (r = -0.29, P<0.29). No significant correlation was found for sleep/wake at T1, T2 or T3 problems and BMI at T1, T2 or T3. However, the growth model was moderated by gender: more sleep problem at 9=higher BMI at 11 (girls only) |
| Fernandez-Mendoza, et al. (2014) ^40^ | Mean WC was significantly (P<0.05) higher among short sleepers (66.1 ± 11.2) compared to normal sleepers (63.5 ± 9.0). BMI percentile did not significantly differ (P = 0.09). Students with parent-reported insomnia symptoms and short sleep duration had a higher mean WC (66.9 ± 13.5)(compared to those with parent-reported insomnia and normal sleep duration (64.7 ± 9.9), P<0.05), this was not significant for BMI percentile. |
| Ferrari et al. (2017) ^41^ | Univariate and multivariate regression analysis found no significant association between self-reported adequate duration or quality of sleep on children's BMI |
| Firouzi et al. (2013) ^42^ | Significant weight status differences were observed for all measures of sleep (bedtime resistant, sleep duration, sleep anxiety, night walking, parasomnias, sleep disordered breathing and daytime sleepiness), P<0.05 except for sleep onset delay N/S. BMI was directly correlated with bedtime (r = 0.15, P<0.05), total sleep disorder score (r =0.41, P<0.001), bedtime resistance (r = 0.24, P<0.01), sleep duration score (r = 0.25, P<0.001), sleep anxiety (r = 0.28, P<0.001), night walking (r = 0.37, P<0.001), parasomnias (r = 0.43, P<0.001) and sleep disordered breathing (r =0.41, P<0.001). |
| García-Hermoso et al. (2017) ^43^ | Sleep quality problem predicted being overweight or obese in girls (OR = 1.41, 95% CI, 1.15 to 1.92, p = 0.001), but not boys. |
| Gentile et al. (2014) ^44^ | In the path model, Average weekly sleep duration at T2 was significantly associated with T2 BMI (B = -0.14, P<0.01). |
| Giovaninni et al. (2014) ^45^ | No significant association between BMI-measured weight status and sleep duration; however, waist circumference and sleep duration ( r = -0.2; P<0.001) |
| Gomes et al. (2014) ^46^ | In the final adjusted multi-level analyses sleep time did not significantly predict BMI. |
| Harrex et al. (2017) ^47^ | No significant differences were found in BMI classification (P = 0.194) or BMI z-score (P = 0.386) across the four sleep timing behaviour groups |
| Hense et al. (2011) ^48^ | In the final adjusted model, compared to those who slept ≥ 11 hrs; those who slept ≤ 9hrs had a higher odds (OR = 3.53; 95% CI: 2.24; 5.54) of being overweight. |
| Hiscock et al. (2011) ^49^ | Cross-sectional analyses found a significant group-level difference in mean sleep duration by weight status for preschool children aged 6-7 years [Underweight = 630.1 mins; Normal weight = 624.9 min; Overweight = 633.9 mins and Obese = 598.0; P<0.001). No significant difference was found for child sleep problems (does your child have a sleep problem, No, Mild, Moderate or Severe Problem) cross-sectionally by weight status. Longitudinal analyses found sleep duration at Wave I did not predict BMI at Wave II, nor did BMI at Wave I predict sleep duration at Wave II. |
| Hjorth et al. (2014) ^50^ | Cross-sectional analyses found FMI (from DXA) was significantly and negatively associated with sleep duration (β **=**-0.74; 95% CI: -1.14; -0.34) in the final adjusted model. Additionally, sleep disturbances was not significantly associated with FMI in the final model. Longitudinally, no significant association was found between sleep duration and FMI. Increases in sleep duration did not decrease FMI, sleep duration at baseline did not significantly predict FMI at follow-up nor did FMI at baseline predict sleep duration at follow-up. Sleep disturbances had no significant influence on FMI Longitudinally. |
| Ievers-Landis et al. (2008) ^51^ | The final adjusted logistical regression model found a 1-hr reduction in parent-reported sleep duration increased the odds of obesity (OR = 1.41. 95%CI: 1.12-1.76). |
| Jiang et al. (2014) ^52^ | Odds ratio of being obese in those who had the shortest sleep duration was 2.27 (95% CI: 1.20 – 4.29) compared with those having the longest sleep. ; with early adolescents in the longest sleep group, those who were in the shortest sleep group still had significantly higher BMI, WHeR and BF% in the adjusted model |
| Jing Jing et al. (2017) ^53^ | Children with shorter sleep durations were significantly more likely to be overweight (AOR: 1.94, 95% CI: 1.36, 2.75). Sleep quality was not significant. |
| Katzmarzyk et al. (2015) ^54^ | OR (95% CI) for obesity according to sleep duration was 0.79 (0.71–0.90) among boys, and 0.71 (0.63–0.80) among girls. |
| Kelly et al. (2016) ^55^ | Non-regular bedtimes led to moderate increasing and high increasing BMI (ORs = 1.22 and 1.55). Late bedtime (after 9pm) was only significantly associated with moderate increased BMI |
| Khan et al. (2015) ^56^ | Longer sleep duration was significantly associated with decreased odds of being overweight or obese (OR = 0.81, 95% CI: 0.75, 0.88); while later bedtimes on weekdays was associated with higher odds of being overweight or obese. |
| Khan et al. (2017) ^57^ | Good sleep quality was associated with a reduced risk of being overweight and obese (ß 0.83, 95%CI:0.76, 0.89; and ß 0.66, 95%CI:0.58, 0.74, respectively); Good sleep duration was negatively associated with being obese (ß -0.16, 95%CI:-0.29, -0.03) |
| Kim et al. (2012) ^58^ | Multivariable logistic regression analyses found increased sleep duration (hr) on weekdays was associated with a lower odds (OR = 0.68; 95% CI: 0.54; 0.86) of being overweight as well as for weekend sleep duration (OR = 0.64; 95% CI: 0.53; 0.77) and catch-up sleep (OR = 0.67; 95% CI: 0.53; 0.85). |
| Kong et al. (2011) ^59^ | Compared to primary school children who slept >9.25hrs; children who slept between 8-9.25hrs had a higher odds of being obese (OR = 2.36: 95%CI: 1.12; 4.98). Additionally, those who slept <8hrs had even greater odds of being obese (OR = 2.88; 95%CI: 1.23; 6.70) compared to those who slept >9.25hrs. |
| Kovács et al. (2015) ^60^ | Sleep duration was associated with an increased risk of overweight/obesity (OR:0.84, 95%CI:0.73–0.98) among school children |
| Krishnan et al. (2017) ^61^ | This study found no evidence of association between increasing sleep duration and reduced obesity risk; although a borderline significant result was detected for BMIz scores (b¼ 0.078 [ 0.158 to 0.0018], p¼ 0.055) |
| Labree et al. (2015) ^62^ | The unstandardized coefficient (B) of sleep duration in the multivariate regression was -0.008 (not in table), implying that, for example, an extra half an hour of sleep, which is about the highest observed mean difference across groups of children, will lead to a BMI decrease of 0.24 (30 minutes -0.008) |
| Larsen et al. (2017) ^63^ | Child sleep duration was negatively associated with child's zBMI for boys, but not for girls (r=-0.28, p<0.01) |
| Laurson et al. (2014) ^64^ | Multivariate models found those who not meet the sleep recommendations had a higher odds of obesity for boys (OR = 2.4; 95% CI: 1.4; 4.3) compared to those that met the recommendations. This association was not significant for girls. |
| Lee et al. (2012) ^65^ | Cross-sectional analyses found significant association with baseline BMI and reported sleep-duration. Longitudinal analyses found 2-year BMI-change was associated with baseline sleep duration, that is, those that reported >9.5hrs of sleep per day had lower BMI increases (B = 0.46; 95%CI: 0.87; 0.54) than those reporting less than 8.5hrs. |
| Lehto et al. (2011) ^66^ | Adjusted models found a significant negative association between sleep (hrs) and WC (β **=** -0.95; 95% CI: -1.77; -0.14) and WHeR (β **=** -0.005; 95%CI: -0.001; -0.00), although this association disappeared in the final adjusted model which adjusted for (age, gender, SB, PA, food intake and BMI) |
| Liu et al. (2011) ^67^ | Compared with those of normal weight, overweight and obese children reported reduced sleep duration (9.4 vs.9.2 hrs, p<0.001), had higher sleep problem scores (16.2 vs.16.8,p =0.048), and waking at night (44.5% vs 55.6%, p=0.014) |
| Lu et al. (2015) ^68^ | Amongst separate rural-to urban migrants, local city and immigrant origin population groups, no significant association was found between sleep duration categories and categorisation of weight status. |
| Lumeng et al. (2007) ^69^ | Adjusted models found sleep duration was associated with a higher odds of overweight (OR = 0.80; 95%CI: 0.65, 0.98). NS night time waking, morning waking and bedtime problems. Longitudinally longer sleep duration in Grade 3 was associated with a lower odds for overweight at Grade 6 (OR = 0.60; 95%CI: 0.36, 0.99). Longitudinal examination of sleep problems at Grade 3 and Grade 6 found no significant relationship with odds for overweight at Grade 6. Bivariate analyses: Bed timing in Grade 6 was significantly later among overweight (21:32 ± 37.2) compared to healthy weight (21:25 ± 38.4), P<0.05). |
| Magee et al. (2013) ^70^ | Bivariate analyses found a significant difference in sleep duration at age 4-5y and growth trajectory [Healthy Trajectory (10.99 ± 0.89h); Early Onset Trajectory (10.71 ± 0.94 h) and Later Onset Trajectory (10.80 ± 1.00h), P<0.05. Further investigations found a cross-sectional relationship in the Healthy Trajectory, Sleep and BMI (β **=**-0.14, P<0.01) at age 10-11y, and sleep and BMI (β **=**-0.61, P<0.05) at age 6 among Early Onset Trajectory and Later Onset Trajectory (β **=** -0.32, P<0.05). Longitudinal relationships with sleep at age 4-5 and BMI at 6-7y (β **=**0.08, P<0.05) among the Healthy Trajectory, Sleep at age 6-7 and BMI age 8-9 (β **=**-0.68, P<0.05) and sleep age 8-9 and BMI age 10-11 (β **=** -1.21, P<0.05) among early Onset Trajectory children. |
| Magee et al. (2013) ^71^ | No significant association between sleep duration and weight status at 6 to 7 years was found; Short sleepers/unhealthy eaters did not have an increased risk of obesity at baseline, but were significantly more likely to be obese at 2-year follow-up (OR = 1.47 (1.03–2.13)) |
| Magee et al. (2014) ^72^ | Results indicated a significant inverse association between sleep duration at 4 years of age and BMI at 8 years of age (β **=** .07, p = .044) |
| Martinez et al. (2014) ^73^ | Sleep duration at baseline was negatively associated with WHeR (B= -0.11, P<0.01), lower weight gain (B = -0.14, P<0.05), and lower BMIz (B = -0.07, P<0.01) after 24 month follow-up. |
| Martinez et al. (2014) ^74^ | Adjusted linear models corroborated this relationship remained significant for mother-reported sleep (b = -0.13; 95%CI: -0.35; -0.04) and accelerometer-measured sleep (b= -0.14; 95%CI: -0.36, -0.03). Insufficient sleep (<10hrs) was associated with a higher odds of overweight/obesity by maternal-report (OR = 2.1; 95%CI: 1.08; 4.24) and was non-significant for accelerometry measured sleep (OR = 1.8; 95%CI: 0.83; 5.1). |
| Martoni et al. (2016) ^75^ | Analysis suggest that obese children (03:18; SD = 00:16) had a phase delay compared to normal weight (03:06; SD = 00:16) and overweight (02:59; SD = 00:14) children (p<0.050, for both comparisons); BMI was also predicted by sleep duration on school days (adjusted R2 = 0.07; F(1,113) = 9.62, p = 0.002). |
| McNeil et al. (2015) ^76^ | Adjusted models found no significant association between sleep duration and WC, BMI-z, %BF, WHeR. Sleep efficiency was significantly association with WC (B= -0.09, P<0.01), BMI-z (B= -0.09, P<0.05); %BF (B= -0.09, P<0.05); and WHtR (B= -0.11, P<0.11). Sleep timing, Bedtime and Wake time were not significantly associated with any measure of adiposity in adjusted models. |
| Meng et al. (2012) ^77^ | Significant negative associations was found in adjusted (including clustered design) between sleep duration and BMI (B = -0.23, P<0.05) and WC (B = -0.82, P<0.01) among boys and BMI (B= -0.24, P<0.01) and WC (B= -0.91, P<0.001) among girls. No significant association with %BF was found. Insufficient sleepers had a higher odds of overweight/obesity (OR = 1.29: 95%CI: 1.01; 1.64) compared to those who slept 10-10.9hrs. |
| Miller (2011) ^78^ | Each additional hour of sleep at Grade 5? was associated with lower BMI (B = 0.06, P<0.01) at baseline (kindergarten) but was not associated with BMI change Longitudinally |
| Morrissey et al. (2016) ^79^ | Adjustment of the initial model for age, gender, SEIFA and study condition shows insufficient sleepers more likely to be categorised as overweight (OR 1.88; 95 % CI:1.14-3.13) or obese (OR 2.31; 95 % CI:1.18-4.53). |
| Munakata et al. (2010) ^80^ | Significant negative correlations with BMI and sleep duration were observed for girls (r = -0.28, P<0.01) but not boys and % body fat for boys (r= -0.22, P<0.05) and girls (r = - 0.25, P< 0.01). |
| Ochiai et al. (2012) ^81^ | In-depth, multivariate analyses found boys who insufficient sleepers (<8.0hrs) had a higher odds for overweight (OR =2.38; 95%CI: 1.05; 5.37) but not for girls. |
| O'Dea et al. (2012) ^82^ | Cross-sectional adjusted analyses found children in the lowest tertile had a higher mean BMI (terile 3 minus tertile 1) for sleep had a in Grade 3 (BMI diff = 1.42; 95%CI: 0.85, 2.00); and Grade 4 (BMI diff = 1.84; 95%CI: 1.21; 2.46), but not Grade 1 or 2. Longitudinally, children in the upper tertile of sleep in Grade 1 had smaller increased in BMI (0.45 ± 0.15 (SE) kg/m2, P<0.004) than children in the lowest sleep tertile in adjusted analyses. |
| Ortega Anta et al. (2013) ^83^ | Bivariate analyses found sleep duration was negatively correlated with BMI (r = -0.30, P<0.001) and WC ( r= - 0.139, P<0.001). Children who were insufficient sleepers (<8hrs) had a higher odds for overweight (OR = 1.67: 95%CI: 1.09; 2.55) and obesity (OR = 2.26: 95%CI: 1.59; 3.20) than those who slept >10hrs/day. |
| Padez et al. (2009) ^84^ | Multivariable logistic regression analyses found that compared to those who slept >= 11hrs; those who slept <9hrs had a higher odds for overweight/obesity (OR = 3.22; 95%CI: 3.11; 3.22). Additionally, those who slept 9-10 hrs (OR =1.16; 95%CI: 1.13; 1.19) and 10-11hrs (OR = 1.3: 95%CI: 1.26, 1.33) had a higher odds for overweight/obesity compared to those who slept >= 11hrs. A significant group-level differences in %FM was also observed among short sleepers [<9 hrs = 23.4%; 9-10hrs = 21.0%; 10-11hrs = 20.5%; >=11hrs = 20.9%], P<0.001. Additionally, bivariate and gender specific association between short sleepers and overweight/obesity was observed for boys only. |
| Peach et al. (2015) ^85^ | Adjusted models found significant negative relationships with a 1hr increase in school-night sleep duration among boys (b= -0.128, P<0.01) and for weekend sleep duration (b= -0.52, P<0.01) (NS for girls). Sleepiness was positively associated with BMI among girls (b= 3.11, P<0.01) and boys (b = 2.55, P<0.05). |
| Pesonen et al. (2009) ^86^ | Children in the bottom decile of sleep quality did not have a significantly higher BMI (16.6 +/- 2.2) compared to the top decile (15.9 +/- 1.9), (P=N/S) |
| Pileggi et al. (2013) ^87^ | Short sleepers had a significantly higher BMI in adjusted models (B= 0.77: 95CI: 0.16; 1.38) compared to normal sleepers. Chronic lack of sleep (not sure how classified - in-text) had a higher odds of overweight/obesity (OR = 1.47; 95%CI: 1.01, 2.12) in adjusted models. |
| Prats-Puig et al. (2013) ^88^ | Adjusted models found a significant negative correlation with BMI (B= -0.6; 95%CI: -1.02; -0.20), WC (B = -1.7; 95%CI: -2.69; -0.54) and visceral fat (B = -0.5; 95%CI: -1.21; -0.30) being negatively associated with sleep duration (hours). |
| Pryor et al. (2015) ^89^ | Children in middle childhood (6-12 years old) who received less than an average of 10 hours of sleep in early childhood (between 2.5-5 years) were reportedly more likely to experience early-onset overweight, compared with never being overweight (OR=1.66, 95%CI: 1.07-2.57), or compared with late-onset overweight (OR=1.68, 95%CI: 1.03-2.75) |
| Quach et al. (2016) ^90^ | As the number of times children had a late-to-sleep profile increased [from 0–1 to 2–3 time points (waves)] there was a significant association with higher child BMI-Z scores, waist circumference. An adjusted linear regression indicated that, compared with Early-to-sleep/Early-to-wake sleepers, those Late-to-sleep/Early-to-wake had higher BMIz scores (though this was only significant among 6-7, and not 8-9 year olds.) |
| Ramos and Barros (2007) ^91^ | Sleep duration had a significant negative association with BMI among males only |
| Reilly et al. (2005) ^92^ | Adjusted models found a significantly higher odds for short sleepers at age 3 to be obese at age 7 (<10.5hrs) (OR = 1.45: 95%CI: 1.10, 1.89)* and those who slept 10.5-10.9hrs (OR = 1.35; 95%CI: 1.02; 1.79)* compared to those who slept >12hrs |
| Rosi et al. (2017) ^93^ | Sleep durations were significantly lower in overweight/obese participants sleep duration (χ2 = 9.7, df = 2, p = 0.008). Sleep timing was not found to differ significantly across weight categories |
| Rudnicka et al. (2017) ^94^ | Longer sleep durations led to lower adjusted means for BMI, sum of skinfolds, fat mass (p<0.001) and fatmass index (p=<0.05). A 1-hour-longer sleep duration was associated with 0.19 lower BMI (95% confidence interval [CI] 0.09 to 0.28), 0.03 kg/m5 lower fat mass index (95% CI 0.00 to 0.05 kg/m5), |
| Santiago et al. (2013) ^95^ | Univariate regression analyses found boys who were long sleepers (>=9hrs) (OR = 0.8: 95%CI: 0.6, 0.9) but not girls had lower odds for overweight/obesity compared to short sleepers (<9hrs). |
| Scharf and DeBoer (2015) ^96^ | Adjusted model found short-sleepers (<9.48hr) did not have a higher odds for overweight or obesity (separately), although later bedtime (after 9:30pm vs before 9:30pm) was significantly associated with an increased odds for overweight (OR = 1.21; 95%CI: 1.00, 1.45) and obesity (OR = 1.49; 95% CI: 1.16, 1.91). Longitudinal adjusted models found sleep (hrs) at age 4 was negatively associate with change in BMI (△) at age 5 [B= -0.03, SE = 0.01]; as well as later bedtime (B = -0.06, SE = 0.02),** but not wake-up time. |
| Sekine et al. (2002) ^97^ | Adjusted analyses found no significant difference in odds for obesity by wake-up time; in contrast a later bedtime was associated with a higher odds of obesity (22:00-23:00) (OR= 1.53; 95%CI: 1.20; 1.95) compared to those who went to bed before 21:00. Sleep duration was also significantly associated with obesity risk with those sleeping <8hr (OR= 3.06; 95%CI: 1.72; 5.36); 8-9h (OR =2.01; 95%CI: 1.43; 2.91) and 9-10h (OR = 1.55; 95%CI: 1.12, 2.22) than those sleeping >= 10hrs. |
| Shah et al. (2013) ^98^ | No significant difference in mean sleep duration was found between normal weight, underweight, overweight or obese children. |
| Silva et al. (2011) ^99^ | Adjusted Longitudinal models found compared to those who slept >=9hrs at baseline, those that slept <7.5hrs had a higher odds for obesity (only) (OR = 3.3; 95%CI: 1.09, 9.66)*. Longitudinal random effects model showed that children who slept <7.5hrs at baseline had an average BMI increase of 1.7 kg/m2 (B= 1.7; 95%CI: 0.4, 0.31)** |
| Stone et al. (2013) ^100^ | Bivariate analyses found a higher proportion of children in the insufficient <9hr category to be overweight/obese on weekdays [<9.5hrs = 36.1%; 9-10hrs = 30.7%; >= 10hrs = 23.6%)*** and weekends [<9hrs = 46.4%; 9-10hrs = 23.7%; and >=10hr = 26.7%]**. |
| Sugimori et al. (2004) ^101^ | Factors at age 6 that are associated with change in BMI from 3 years of age to 6 years of age. Bedtime (10pm or later) did not significantly differ by weight status change (Norm to Norm), (Obese to Norm), (Norm to Obese), (Obese to Obese), nor did wake time (before 7am) among boys. However, group-level differences in the proportion sleeping <9hrs did relate to weight status category change with 21.8% (Norm to Norm), 29.2% (Obese to Norm), 33.9% (Norm to Obese), 36.7% (Obese to Obese)**** getting 9hrs of sleep or less at 6 years of age among boys. Among girls, bedtime (10pm or later) was associated with weight status change (89.9% Norm to Norm), (90.8% Obese to Norm), (82.1% Norm to Obese) and 86.4% (obese to Obese)***; No significant duration difference was evident for girls |
| Suglia et al. (2013) ^102^ | Short sleep duration was also associated with obesity among girls (PR, 1.2; 95% CI, 1.0–1.4) and boys (PR, 1.3; 95% CI, 1.1–1.5) |
| Sun et al. (2009) ^103^ | Sleep duration did not relate to overweight risk in adjusted models among males. Among females, those who slept <7hrs had a higher odds for overweight (OR =1.81; 95%CI: 1.21, 2.71) and 7-8hrs (OR = 1.37; 95% CI: 1.00, 1.88) compared to those who slept 8-9hrs. No significant association between either rising time or bedtime and overweight was found for either sex in the 3 models |
| Taveras et al. (2014) ^104^ | Children with a sleep score of 0 to 4 had a higher BMIz (B = 0.48;95%CI: 0.13, 0.83), sub scapular skinfold (B = 4.22; 95%CI: 0.72; 7.72), total FMI (B=0.72, 95%CI: 0.02, 1.42), trunk FMI (b= 0.36; 95%CI: 0.05, 0.67), WC (b= 3.61; 95%CI: 0.74, 6.48), hip circumference (b= 2.78; 95%CI: 0.23, 5.32) and odds of obesity (OR = 2.62: 95%CI: 0.99, 6.97) than those who had a sleep score of 12-13. |
| Thasanasuwan et al. (2016) ^105^ | More than double the risk of being obese was found amongst those who slept less than 9 hours per day, compared with >10 |
| Thivel et al. (2015) ^106^ | Late sleepers compared to normal sleepers had a higher FM% (19.52 +/- 7.44 vs 17.44 +/- 6.23)* and WC (60.1 +/- 7.6 vs 58.2 +/- 4.9)* (BMI and Skinfolds N/S) respectively. |
| Tovaret al. (2012) ^107^ | Bivariate analyses found significant group-level differences in the proportion of children classified as healthy weight (76.1 vs 23.9%), overweight (48.4 vs 51.9%) and obese (69.7% vs 30.3%) between insufficient and sufficient sleepers, (P<0.05). In adjusted models, those with sufficient sleep had a higher odds of overweight (OR = 3.7; 95%CI: 1.97, 7.2). |
| Tuyet et al. (2017) ^108^ | Obesity risk was significantly associated with category of night sleep duration (<8h/day vs. >8h/day, OR=2.56 (95%CI:1.53–4.29), p< 0.0001) |
| Von Kries et al. (2002) ^109^ | Adjusted odds ratio for overweight compared to insufficient sleepers (<=10hrs) was lower for obesity for sleeping 10.5-11hr (OR = 0.52; 95%CI: 0.34, 0.78). Compared to insufficient sleepers (<= 10hrs) those who slept longer (10.5-11hrs) had a lower odds for overweight (OR = 0.77; 95%CI: 0.59, 0.99), obesity (OR = 0.53; 95%CI: 0.35, 0.80) and high body fat (OR = 0.61; 95%CI: 0.26, 0.99). This was also observed among those who slept >= 11hrs for overweight (OR = 0.54; 95%CI: 0.40, 0.73), obese (OR = 0.45; 95%CI: 0.28, 0.75) and high body fat (OR = 0.42; 95%CI: 0.24, 0.76). |
| Wang et al. (2016) ^110^ | sleeping less than 10 h was remarkably associated with a higher risk of overweight (adjusted RR [95% CI]: 1.48 1.26–1.74) and obesity (adjusted RR [95% CI]: 1.77, 1.30–2.40) compared with sleeping 11–12 h |
| Wang et al. (2017) ^111^ | Longer sleep duration was inversely associated with BMI z-score (b ¼0.16, p < 0.05), WC (b ¼1.11, p < 0.05) and later bedtime was associated with higher BMI z-score (b ¼ 0.03, p < 0.05), WC (b ¼ 1.72, p < 0.001), and BF% (b ¼ 0.15, p < 0.05); no significant association was found between sleep quality and adiposity |
| Wells et al. (2008) ^112^ | Adjusted analyses found each additional hour of sleep was associated with lower BMI (B = 0.16, SE = 0.04), triceps skinfold (B= -0.16, SE = 0.07) and subscapular skinfold (B = -0.17, SE = 0.06) (all P<0.05). which was associated with a higher odds for obesity (OR = 0.86, SE = 0.04) all P<0.001 |
| Wijnhoven et al. (2015) ^113^ | Multivariate analysis found no significant association of sleep duration on weight status across the studied countries. Only Portugal and Sweden presented significant in the univariate analysis |
| Williams et al. (2013) ^114^ | Sleep time at age 5 was negatively associated with BMI at age 7 (B = -0.09; 95%CI: -0.17, 0.00), P<0.05). |
| Wong et al. (2013) ^115^ | In adjusted models, obese children slept less (β **=** -0.2hrs, p<0.02) than normal weight children. An additional increase in BMI z-score was associated with a reduction in sleep (-0.1 hr/d, P<0.001). |
| Zhang et al. (2016) | Sleep duration of 9-11 hours (versus 7-8.9 hours) was negatively associated with overweight (adjusted OR: 0.60; 95 % CI: 0.43–0.84), but not with obesity. |
| Key: **BF**= Body Fat; **FMI**= Fat Mass Index, **WHeR**=waist/height ratio, **SB**=sedentary behaviour, **PA**=physical activity, **NEG**= negative association reported; **POS**= positive association reported; **Sig** = Significant association; **NS**= no significant association reported; **CS**=cross-sectional; **LT**= longitudinal | |

References

1. Cappuccio FP, Taggart FM, Kandala N-B, et al. Meta-analysis of short sleep duration and obesity in children and adults. *Sleep.* 2008;31(5):619-626.

2. Chen X, Beydoun MA, Youfa W. Is Sleep Duration Associated With Childhood Obesity? A Systematic Review and Meta-analysis. *Obesity (19307381).* 2008;16(2):265-274.

3. Fatima Y, Doi SAR, Mamun AA. Longitudinal impact of sleep on overweight and obesity in children and adolescents: a systematic review and bias-adjusted meta-analysis. *Obesity Reviews.* 2015:n/a-n/a.

4. Liu J, Zhang A, Li L. Sleep duration and overweight/obesity in children: review and implications for pediatric nursing. *Journal For Specialists In Pediatric Nursing: JSPN.* 2012;17(3):193-204.

5. Magee L, Hale L. Longitudinal associations between sleep duration and subsequent weight gain: A systematic review. *Sleep Medicine Reviews.* 2012;16(3):231-241.

6. Patel SR, Hu FB. Short sleep duration and weight gain: a systematic review. *Obesity.* 2008;16(3):643-653.

7. Agras WS, Hammer LD, McNicholas F, Kraemer HC. Risk factors for childhood overweight: a prospective study from birth to 9.5 years. *The Journal Of Pediatrics.* 2004;145(1):20-25.

8. Alamian A, Wang L, Hall AM, Pitts M, Ikekwere J. Infant sleep problems and childhood overweight: Effects of three definitions of sleep problems. *Preventive Medicine Reports.* 2016;4:463-468.

9. Alqaderi H, Redline S, Tavares M, Goodson JM. Effect of late bedtime on salivary glucose and abdominal obesity in children. *Sleep and Biological Rhythms.* 2017;15(3):227-233.

10. Altenburg TM, Chinapaw MJM, van der Knaap ETW, Brug J, Manios Y, Singh AS. Longer Sleep - Slimmer Kids: The ENERGY-Project. *PLoS ONE.* 2013;8(3).

11. Amigo I, Pena E, Errasti JM, Busto R. Sedentary versus active leisure activities and their relationship with sleeping habits and body mass index in children of 9 and 10 years of age. *Journal of health psychology.* 2014.

12. Arora T, Taheri S. Associations among late chronotype, body mass index and dietary behaviors in young adolescents. *International Journal of Obesity.* 2015;39(1):39-44.

13. Bagley EJ, El-Sheikh M. Familial risk moderates the association between sleep and zBMI in children. *Journal of Pediatric Psychology.* 2013;38(7):775-784.

14. Bagley EJ, El-Sheikh M. Relations between daytime pre-ejection period reactivity and sleep in late childhood. *Journal of sleep research.* 2014;23(3):335-338.

15. Barlett ND, Gentile DA, Barlett CP, Eisenmann JC, Walsh DA. Sleep as a mediator of screen time effects on US children's health outcomes: A prospective study. *Journal of Children and Media.* 2012;6(1):37-50.

16. Bayer O, Rosario AS, Wabitsch M, von Kries R. Sleep duration and obesity in children: Is the association dependent on age and choice of the outcome parameter? *Sleep: Journal of Sleep and Sleep Disorders Research.* 2009;32(9):1183-1189.

17. Bell JF, Zimmerman FJ. Shortened nighttime sleep duration in early life and subsequent childhood obesity. *Archives of Pediatrics and Adolescent Medicine.* 2010;164(9):840-845.

18. Berentzen NE, Smit HA, Bekkers MBM, et al. Time in bed, sleep quality and associations with cardiometabolic markers in children: The prevention and incidence of asthma and mite allergy birth cohort study. *Journal of sleep research.* 2014;23(1):3-12.

19. Busto-Zapico R, Amigo-Vazquez I, Pena-Suarez E, Fernandez-Rodriguez C. Relationships between sleeping habits, sedentary leisure activities and childhood overweight and obesity. *Psychol Health Med.* 2014;19(6):667-672.

20. Cameron AJ, van Stralen MM, Brug J, et al. Television in the bedroom and increased body weight: Potential explanations for their relationship among European schoolchildren. *Pediatric Obesity.* 2013;8(2):130-141.

21. Cao M, Zhu Y, He B, et al. Association between sleep duration and obesity is age- and gender-dependent in Chinese urban children aged 6-18 years: a cross-sectional study. *BMC Public Health.* 2015;15:1029.

22. Carrillo-Larco RM, Bernabe-Ortiz A, Miranda JJ. Short sleep duration and childhood obesity: Cross-sectional analysis in Peru and patterns in four developing countries. *PLoS ONE.* 2014;9(11).

23. Carter PJ, Taylor BJ, Williams SM, Taylor RW. Longitudinal analysis of sleep in relation to BMI and body fat in children: The FLAME study. *BMJ: British Medical Journal.* 2011;342(7809):1-7.

24. Casazza K, Hanks LJ, Fernandez JR. Shorter Sleep may be a Risk Factor for Impaired Bone Mass Accrual in Childhood. *Journal of Clinical Densitometry.* 2011;14(4):453-457.

25. Cassimos D, Sidiropoulos H, Batzios S, Balodima V, Christoforidis A. Sociodemographic and dietary risk factors for excess weight in a greek pediatric population living in Kavala, Northern Greece. *Nutrition in Clinical Practice.* 2011;26(2):186-191.

26. Chahal H, Fung C, Kuhle S, Veugelers PJ. Availability and night-time use of electronic entertainment and communication devices are associated with short sleep duration and obesity among Canadian children. *Pediatric Obesity.* 2013;8(1):42-51.

27. Chaput JP, Brunet M, Tremblay A. Relationship between short sleeping hours and childhood overweight/obesity: Results from the 'Quebec en Forme' project. *International Journal of Obesity.* 2006;30(7):1080-1085.

28. Colley RC, Wong SL, Garriguet D, Janssen I, Connor Gorber S, Tremblay MS. Physical activity, sedentary behaviour and sleep in Canadian children: parent-report versus direct measures and relative associations with health risk. *Health Reports.* 2012;23(2):45-52.

29. Combs D, Goodwin JL, Quan SF, Morgan WJ, Parthasarathy S. Longitudinal differences in sleep duration in Hispanic and Caucasian children. *Sleep Medicine.* 2016;18:61-66.

30. de Jong E, Stocks T, Visscher TLS, HiraSing RA, Seidell JC, Renders CM. Association between sleep duration and overweight: the importance of parenting. *International Journal of Obesity.* 2012;36(10):1278-1284.

31. Del Pozo-Cruz B, Gant N, Del Pozo-Cruz J, Maddison R. Relationships between sleep duration, physical activity and body mass index in young New Zealanders: An isotemporal substitution analysis. *PLoS ONE.* 2017;12(9).

32. Diethelm K, Bolzenius K, Cheng G, Remer T, Buyken AE. Longitudinal associations between reported sleep duration in early childhood and the development of body mass index, fat mass index and fat free mass index until age 7. *International Journal of Pediatric Obesity.* 2011;6(2-2):e114-e123.

33. Drescher AA, Goodwin JL, Silva GE, Quan SF. Caffeine and screen time in adolescence: Associations with short sleep and obesity. *Journal of Clinical Sleep Medicine.* 2011;7(4):337-342.

34. Duncan JS, Schofield G, Duncan EK, Rush EC. Risk factors for excess body fatness in New Zealand children. *Asia Pacific journal of clinical nutrition.* 2008;17(1):138-147.

35. Duran Aguero S, Haro Rivera P. Association between the amount of sleep and obesity in Chilean schoolchildren. *Archivos argentinos de pediatria.* 2016;114(2):114-119.

36. Eisenmann JC, Ekkekakis P, Holmes M. Sleep duration and overweight among Australian children and adolescents. *Acta Paediatrica (Oslo, Norway: 1992).* 2006;95(8):956-963.

37. Ekstedt M, Nyberg G, Ingre M, Ekblom Ö, Marcus C. Sleep, physical activity and BMI in six to ten-year- old children measured by accelerometry: A cross-sectional study. *The International Journal of Behavioral Nutrition and Physical Activity.* 2013;10.

38. El-Sheikh M, Erath SA, Keller PS. Children's sleep and adjustment: The moderating role of vagal regulation. *Journal of sleep research.* 2007;16(4):396-405.

39. El-Sheikh M, Bagley EJ, Keiley MK, Erath SA. Growth in body mass index from childhood into adolescence: The role of sleep duration and quality. *The Journal of Early Adolescence.* 2014;34(8):1145-1166.

40. Fernandez-Mendoza J, Vgontzas AN, Calhoun SL, et al. Insomnia symptoms, objective sleep duration and hypothalamic-pituitary-adrenal activity in children. *European Journal of Clinical Investigation.* 2014;44(5):493-500.

41. Ferrari GLDM, Matsudo V, Katzmarzyk PT, Fisberg M. Prevalence and factors associated with body mass index in children aged 9–11 years. *J Pediatr (Rio J).* 2017;93(6):601-609.

42. Firouzi S, Koon PB, Noor MI, Sadeghilar A. Sleep pattern and sleep disorders among a sample of Malaysian children. *Sleep and Biological Rhythms.* 2013;11(3):185-193.

43. García-Hermoso A, Aguilar MM, Vergara FA, Velásquez EJA, Marina R. Obesity, cardiorespiratory fitness, and self-reported sleep patterns in Chilean school-aged children. *Behavioral Sleep Medicine.* 2017;15(1):70-80.

44. Gentile DA, Reimer RA, Nathanson AI, Walsh DA, Eisenmann JC. Protective effects of parental monitoring of children's media use a prospective study. *JAMA Pediatrics.* 2014;168(5):479-484.

45. Giovaninni NP, Fuly JT, Moraes LI, et al. Study of the association between 3111T/C polymorphism of the CLOCK gene and the presence of overweight in schoolchildren. *Jornal de Pediatria.* 2014;90(5):500-505.

46. Gomes TN, Katzmarzyk PT, dos Santos FK, Souza M, Pereira S, Maia JAR. Overweight and obesity in Portuguese children: prevalence and correlates. *International Journal Of Environmental Research And Public Health.* 2014;11(11):11398-11417.

47. Harrex HAL, Skeaff SA, Black KE, et al. Sleep timing is associated with diet and physical activity levels in 9–11‐year‐old children from dunedin, new zealand: The pedals study. *Journal of sleep research.* 2017.

48. Hense S, Pohlabeln H, De Henauw S, et al. Sleep duration and overweight in European children: Is the association modified by geographic region? *Sleep: Journal of Sleep and Sleep Disorders Research.* 2011;34(7):885-890.

49. Hiscock H, Scalzo K, Canterford L, Wake M. Sleep duration and body mass index in 0-7-year olds. *Archives of Disease in Childhood.* 2011;96(8):735-739.

50. Hjorth MF, Chaput JP, Ritz C, et al. Fatness predicts decreased physical activity and increased sedentary time, but not vice versa: Support from a longitudinal study in 8- to 11-year-old children. *International Journal of Obesity.* 2014;38(7):959-965.

51. Ievers-Landis CE, Storfer-Isser A, Rosen C, Johnson NL, Redline S. Relationship of sleep parameters, child psychological functioning, and parenting stress to obesity status among preadolescent children. *Journal of Developmental and Behavioral Pediatrics.* 2008;29(4):243-252.

52. Jiang YR, Spruyt K, Chen WJ, et al. Associations between parent-reported sleep duration and adiposity in Chinese early adolescents. *Journal Of Public Health (Oxford, England).* 2014.

53. Jing Jing W, Yang G, Lau PWC. Prevalence of overweight in Hong Kong Chinese children: Its associations with family, early-life development and behaviors-related factors. *Journal of Exercise Science & Fitness.* 2017;15(2):89-95.

54. Katzmarzyk PT, Barreira TV, Broyles ST, et al. Relationship between lifestyle behaviors and obesity in children ages 9-11: Results from a 12-country study. *Obesity (19307381).* 2015;23(8):1696-1702.

55. Kelly Y, Patalay P, Montgomery S, Sacker A. BMI Development and Early Adolescent Psychosocial Well-Being: UK Millennium Cohort Study. *Pediatrics.* 2016;138(6):84-84.

56. Khan MKA, Yen Li C, Kirk SFL, Veugelers PJ, Chu YL. Are sleep duration and sleep quality associated with diet quality, physical activity, and body weight status? A population-based study of Canadian children. *Canadian Journal of Public Health.* 2015;106(5):e277-e282.

57. Khan MKA, Faught EL, Chu YL, Ekwaru JP, Storey KE, Veugelers PJ. Is it nutrients, food items, diet quality or eating behaviours that are responsible for the association of children's diet with sleep? *Journal of sleep research.* 2017;26(4):468-476.

58. Kim C-W, Choi M-K, Im H-J, et al. Weekend catch-up sleep is associated with decreased risk of being overweight among fifth-grade students with short sleep duration. *Journal of sleep research.* 2012;21(5):546-551.

59. Kong AP, Wing Y-K, Choi KC, et al. Associations of sleep duration with obesity and serum lipid profile in children and adolescents. *Sleep Medicine.* 2011;12(7):659-665.

60. Kovács E, Hunsberger M, Reisch L, et al. Adherence to combined lifestyle factors and their contribution to obesity in the IDEFICS study. *Obesity Reviews.* 2015;16:138-150.

61. Krishnan M, Shelling AN, McCowan LME, et al. Gene-by-environment interactions of the CLOCK, PEMT, and GHRELIN loci with average sleep duration in relation to obesity traits using a cohort of 643 New Zealand European children. *Sleep Medicine.* 2017;37:19-26.

62. Labree W, van de Mheen D, Rutten F, Rodenburg G, Koopmans G, Foets M. Differences in Overweight and Obesity among Children from Migrant and Native Origin: The Role of Physical Activity, Dietary Intake, and Sleep Duration. *PLoS One.* 2015;10(6):e0123672.

63. Larsen JK, Sleddens EFC, Vink JM, van den Broek N, Kremers SPJ. The sex-specific interaction between food responsiveness and sleep duration explaining body mass index among children. *Sleep Medicine.* 2017;40:106-109.

64. Laurson KR, Lee JA, Gentile DA, Walsh DA, Eisenmann JC. Concurrent Associations between Physical Activity, Screen Time, and Sleep Duration with Childhood Obesity. *ISRN Obesity.* 2014;2014:204540-204540.

65. Lee HH, Park HA, Kang JH, et al. Factors related to body mass index and body mass index change in korean children: preliminary results from the obesity and metabolic disorders cohort in childhood. *Korean Journal Of Family Medicine.* 2012;33(3):134-143.

66. Lehto R, Ray C, Lahti-Koski M, Roos E. Health behaviors, waist circumference and waist-to-height ratio in children. *Eur J Clin Nutr.* 2011;65(7):841-848.

67. Liu J, Hay J, Joshi D, Faught BE, Wade T, Cairney J. Sleep difficulties and obesity among preadolescents. *Canadian Journal of Public Health.* 2011;102(2):139-143.

68. Lu JK, Yin XJ, Xiong JP, Liu JJ, Watanabe T, Tanaka T. Comparison of the status of overweight/obesity among the youth of local Shanghai, young rural-to-urban migrants and immigrant origin areas. *International Journal of Clinical and Experimental Medicine.* 2015;8(2):2804-2814.

69. Lumeng JC, Somashekar D, Appugliese D, Kaciroti N, Corwyn RF, Bradley RH. Shorter sleep duration is associated with increased risk for being overweight at ages 9 to 12 years. *Pediatrics.* 2007;120(5):1020-1029.

70. Magee CA, Caputi P, Iverson DC. The longitudinal relationship between sleep duration and body mass index in children: A growth mixture modeling approach. *Journal of Developmental and Behavioral Pediatrics.* 2013;34(3):165-173.

71. Magee CA, Caputi P, Iverson DC. Patterns of health behaviours predict obesity in Australian children. *Journal of Paediatrics and Child Health.* 2013;49(4):291-296.

72. Magee C, Caputi P, Iverson D. Lack of sleep could increase obesity in children and too much television could be partly to blame. *Acta Paediatrica.* 2014;103(1):e27-e31.

73. Martinez SM, Tschann JM, Greenspan LC, et al. Is it time for bed? Short sleep duration increases risk of obesity in Mexican American children. *Sleep Medicine.* 2014;15(12):1484-1489.

74. Martinez SM, Greenspan LC, Butte NF, et al. Mother-reported sleep, accelerometer-estimated sleep and weight status in Mexican American children: Sleep duration is associated with increased adiposity and risk for overweight/obese status. *Journal of sleep research.* 2014;23(3):326-334.

75. Martoni M, Carissimi A, Fabbri M, Filardi M, Tonetti L, Natale V. 24-h actigraphic monitoring of motor activity, sleeping and eating behaviors in underweight, normal weight, overweight and obese children. *Eating and Weight Disorders.* 2016;21(4):669-677.

76. McNeil J, Tremblay MS, Leduc G, et al. Objectively-measured sleep and its association with adiposity and physical activity in a sample of Canadian children. *Journal of sleep research.* 2014.

77. Meng LP, Liu AL, Hu X, et al. Report on childhood obesity in China (10): association of sleep duration with obesity. *Biomedical And Environmental Sciences: BES.* 2012;25(2):133-140.

78. Miller DP. Associations between the home and school environments and child body mass index. *Social Science & Medicine.* 2011;72(5):677-684.

79. Morrissey B, Malakellis M, Whelan J, et al. Sleep duration and risk of obesity among a sample of Victorian school children. *BMC Public Health.* 2016;16(1):1-8.

80. Munakata H, Sei M, Ewis AA, et al. Prediction of Japanese children at risk for complications of childhood obesity: Gender differences for intervention approaches. *Journal of Medical Investigation.* 2010;57(1-2):62-68.

81. Ochiai H, Shirasawa T, Shimada N, et al. Sleep duration and overweight among elementary schoolchildren: A population-based study in Japan. *Acta Medica Okayama.* 2012;66(2):93-99.

82. O'Dea JA, Dibley MJ, Rankin NM. Low sleep and low socioeconomic status predict high body mass index: A 4-year longitudinal study of Australian schoolchildren. *Pediatric Obesity.* 2012;7(4):295-303.

83. Ortega Anta RM, López-Solaber AM, Pérez-Farinós N. Associated factors of obesity in Spanish representative samples. *Nutrición Hospitalaria.* 2013;28 Suppl 5:56-62.

84. Padez C, Mourao I, Moreira P, Rosado V. Long sleep duration and childhood overweight/obesity and body fat. *American Journal of Human Biology.* 2009;21(3):371-376.

85. Peach H, Gaultney JF, Reeve CL. Sleep characteristics, body mass index, and risk for hypertension in young adolescents. *Journal of Youth and Adolescence.* 2015;44(2):271-284.

86. Pesonen A-K, Räikkönen K, Matthews K, et al. Prenatal origins of poor sleep in children. *Sleep: Journal of Sleep and Sleep Disorders Research.* 2009;32(8):1086-1092.

87. Pileggi C, Lotito F, Bianco A, Nobile CG, Pavia M. Relationship between Chronic Short Sleep Duration and Childhood Body Mass Index: A School-Based Cross-Sectional Study. *PLoS One.* 2013;8(6):e66680.

88. Prats-Puig A, Grau-Cabrera P, Riera-Pérez E, et al. Variations in the obesity genes FTO, TMEM18 and NRXN3 influence the vulnerability of children to weight gain induced by short sleep duration. *International Journal Of Obesity (2005).* 2013;37(2):182-187.

89. Pryor LE, Brendgen M, Tremblay RE, et al. Early risk factors of overweight developmental trajectories during middle childhood. *PLoS ONE.* 2015;10(6).

90. Quach J, Price AMH, Bittman M, Hiscock H. Sleep timing and child and parent outcomes in Australian 4–9-year-olds: A cross-sectional and longitudinal study. *Sleep Medicine.* 2016;22:39-46.

91. Ramos E, Barros H. Family and school determinants of overweight in 13-year-old Portuguese adolescents. *Acta Paediatrica.* 2007;96(2):281-286.

92. Reilly JJ, Armstrong J, Dorosty AR, et al. Early life risk factors for obesity in childhood: Cohort study. *BMJ: British Medical Journal.* 2005;330(7504):1357-1357.

93. Rosi A, Calestani MV, Parrino L, et al. Weight status is related with gender and sleep duration but not with dietary habits and physical activity in primary school Italian children. *Nutrients.* 2017;9(6):1-10.

94. Rudnicka AR, Nightingale CM, Donin AS, et al. Sleep Duration and Risk of Type 2 Diabetes. *Pediatrics.* 2017;140(3):1-10.

95. Santiago S, Zazpe I, Martí A, Cuervo M, Martínez JA. Gender differences in lifestyle determinants of overweight prevalence in a sample of Southern European children. *Obesity Research & Clinical Practice.* 2013;7(5):e391-e400.

96. Scharf RJ, DeBoer MD. Sleep timing and longitudinal weight gain in 4- and 5-year-old children. *Pediatric Obesity.* 2015;10(2):141-148.

97. Sekine M, Yamagami T, Handa K, et al. A dose-response relationship between short sleeping hours and childhood obesity: Results of the Toyama birth cohort study. *Child: Care, Health and Development.* 2002;28(2):163-170.

98. Shah JS, Patel PK, Patel B. Determinants of overweight and obesity among school children in Mehsana District, India. *Annals of Tropical Medicine & Public Health.* 2013;6(4):408-412.

99. Silva GE, Goodwin JL, Parthasarathy S, et al. Longitudinal association between short sleep, body weight, and emotional and learning problems in Hispanic and Caucasian children. *Sleep: Journal of Sleep and Sleep Disorders Research.* 2011;34(9):1197-1205.

100. Stone MR, Stevens D, Faulkner GEJ. Maintaining recommended sleep throughout the week is associated with increased physical activity in children. *Preventive Medicine: An International Journal Devoted to Practice and Theory.* 2013;56(2):112-117.

101. Sugimori H, Yoshida K, Izuno T, et al. Analysis of factors that influence body mass index from ages 3 to 6 years: A study based on the Toyama cohort study. *Pediatrics International.* 2004;46(3):302-310.

102. Suglia SF, Duarte CS, Chambers EC, Boynton-Jarrett R. Social and behavioral risk factors for obesity in early childhood. *Journal of Developmental and Behavioral Pediatrics.* 2013;34(8):549-556.

103. Sun Y, Sekine M, Kagamimori S. Lifestyle and overweight among Japanese adolescents: the Toyama Birth Cohort Study. *Journal of epidemiology / Japan Epidemiological Association.* 2009;19(6):303-310.

104. Taveras EM, Gillman MW, Peña M-M, Redline S, Rifas-Shiman SL. Chronic sleep curtailment and adiposity. *Pediatrics.* 2014;133(6):1013-1022.

105. Thasanasuwan W, Srichan W, Kijboonchoo K, et al. Low sleeping time, high TV viewing time, and physical inactivity in school are risk factors for obesity in pre-adolescent Thai children. *Journal of the Medical Association of Thailand.* 2016;99(3):314-321.

106. Thivel D, Isacco L, Aucouturier J, et al. Bedtime and Sleep Timing but not Sleep Duration Are Associated With Eating Habits in Primary School Children. *Journal Of Developmental And Behavioral Pediatrics: JDBP.* 2015.

107. Tovar A, Chui K, Hyatt RR, et al. Healthy-lifestyle behaviors associated with overweight and obesity in US rural children. *BMC Pediatrics.* 2012;12.

108. Tuyet LT, Nhung BT, Dao DTA, et al. The Brain-Derived Neurotrophic Factor Val66Met Polymorphism, Delivery Method, Birth Weight, and Night Sleep Duration as Determinants of Obesity in Vietnamese Children of Primary School Age. *Childhood Obesity.* 2017;13(5):392-399.

109. Von Kries R, Toschke AM, Wurmser H, Sauerwald T, Koletzko B. Reduced risk for overweight and obesity in 5- and 6-y-old children by duration of sleep - A cross-sectional study. *International Journal of Obesity.* 2002;26(5):710-716.

110. Wang F, Liu H, Wan Y, et al. Sleep duration and overweight/obesity in preschool-aged children: A prospective study of up to 48,922 children of the Jiaxing Birth Cohort. *Sleep: Journal of Sleep and Sleep Disorders Research.* 2016;39(11):2013-2019.

111. Wang J, Adab P, Liu W, et al. Prevalence of adiposity and its association with sleep duration, quality, and timing among 9-12-year-old children in Guangzhou, China. *Journal of epidemiology.* 2017;27(11):531-537.

112. Wells JCK, Hallal PC, Reichert FF, Menezes AMB, Araújo CLP, Victora CG. Sleep patterns and television viewing in relation to obesity and blood pressure: evidence from an adolescent Brazilian birth cohort. *International Journal of Obesity.* 2008;32(7):1042-1049.

113. Wijnhoven TM, van Raaij JM, Yngve A, et al. WHO European Childhood Obesity Surveillance Initiative: health-risk behaviours on nutrition and physical activity in 6-9-year-old schoolchildren. *Public Health Nutrition.* 2015;18(17):3108-3124.

114. Williams SM, Taylor RW, Taylor BJ. Secular changes in BMI and the associations between risk factors and BMI in children born 29 years apart. *Pediatric Obesity.* 2013;8(1):21-30.

115. Wong WW, Ortiz CL, Lathan D, et al. Sleep duration of underserved minority children in a cross-sectional study. *BMC Public Health.* 2013;13:648-648.
